# Supplementary material for: Dormant pathogenic CD4+ T cells are prevalent in the peripheral repertoire of healthy mice
Source: Nat Commun. 2019 Oct 25;10:4882. doi: 10.1038/s41467-019-12820-3 (PMC6814812; doi:10.1038/s41467-019-12820-3)
Supplement: Supplementary file 1 — Supplementary Information [file 41467_2019_12820_MOESM1_ESM.pdf]

Supplementary Information

# Dormant pathogenic CD4$^+$Foxp3$^-$ T cells are prevalent in the peripheral repertoire of healthy mice.

A. Cebula et al.

**Supplementary Figure 1**

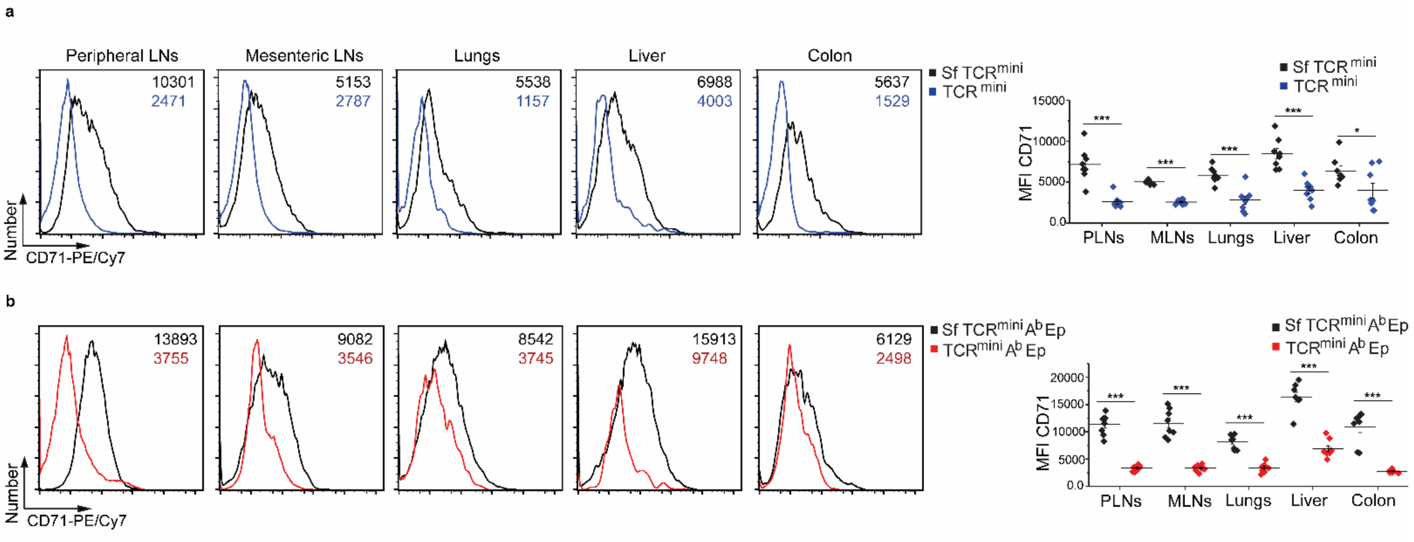


**Supplementary Figure 1. a Expression of CD71 on CD4$^+$CD44$^+$CD62L$^-$ T cells from SfTCR$^{mini}$ and control TCR$^{mini}$ mice.** Histograms show representative expression of indicated molecule *ex vivo*, and the graph shows the summary result. **b Expression of CD71 on CD4$^+$CD44$^+$CD62L$^-$ cells isolated from SfTCR$^{mini}$A$^b$Ep and control TCR$^{mini}$A$^b$Ep mice.** Histograms depict representative expression levels of CD71 by CD4$^+$Foxp3$^{GFP-}$ T cells isolated from indicated organs from SfTCR$^{mini}$A$^b$Ep or control TCR$^{mini}$A$^b$Ep stains, and the graph shows a summary result. Unpaired t-test was applied, and statistical significance is indicated where appropriate (* $p<0.05$, ** $p<0.01$, *** $p<0.001$).

**Supplementary Figure 2**

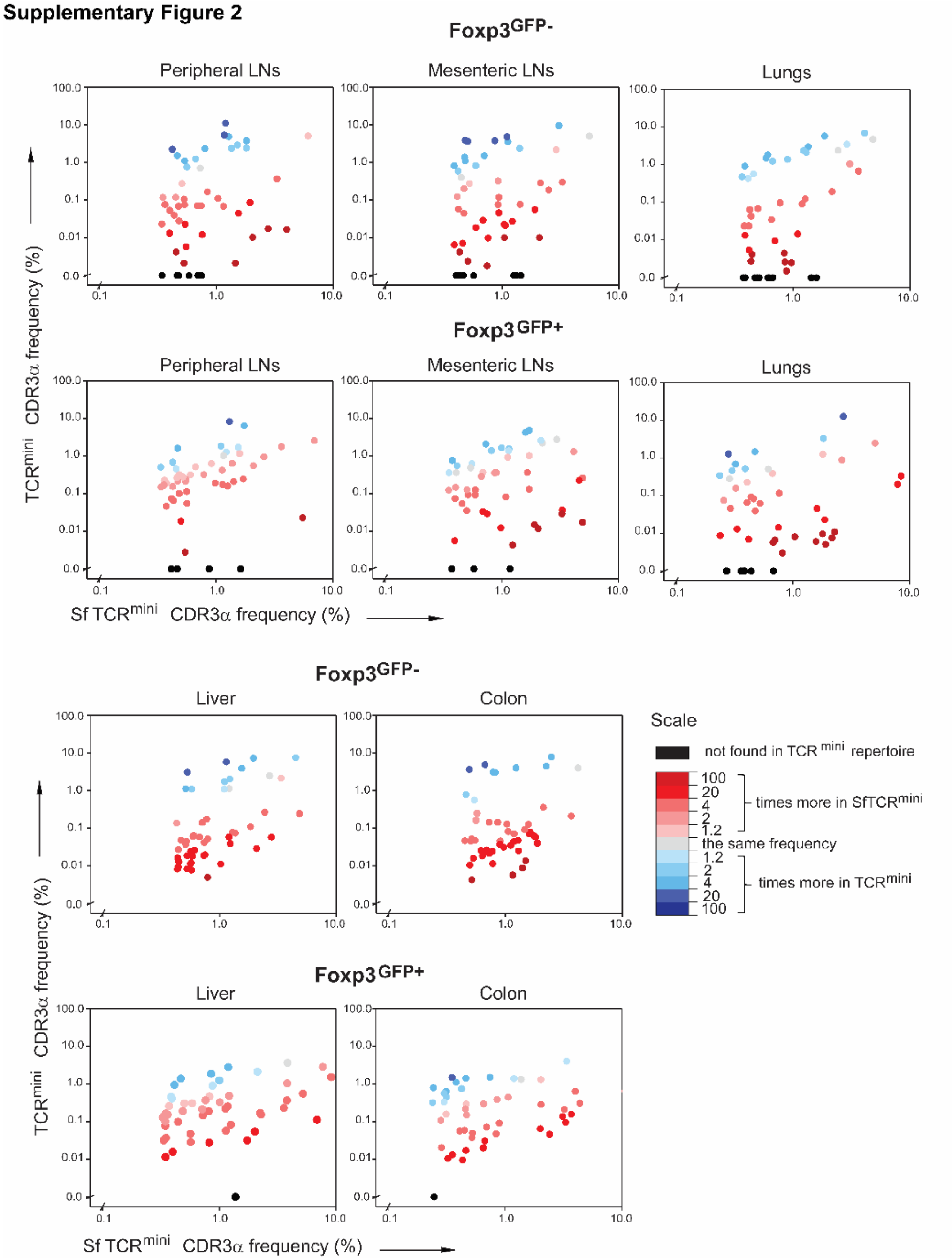


**Supplementary Figure 2. Frequencies of the 50 dominant TCRs from SfCD4$^{+}$ cells in control TCR$^{mini}$ mice.** Color scale shows fold change in frequencies of identical TCRs overrepresented in SfTCR$^{mini}$ (red dots) vs TCR$^{mini}$ repertoires (blue dots). Black dots mark TCRs found exclusively in SfTCR$^{mini}$ repertoires. Repertoires from different organs of 3 individual mice from each examined strain are shown.

## Supplementary Figure 3

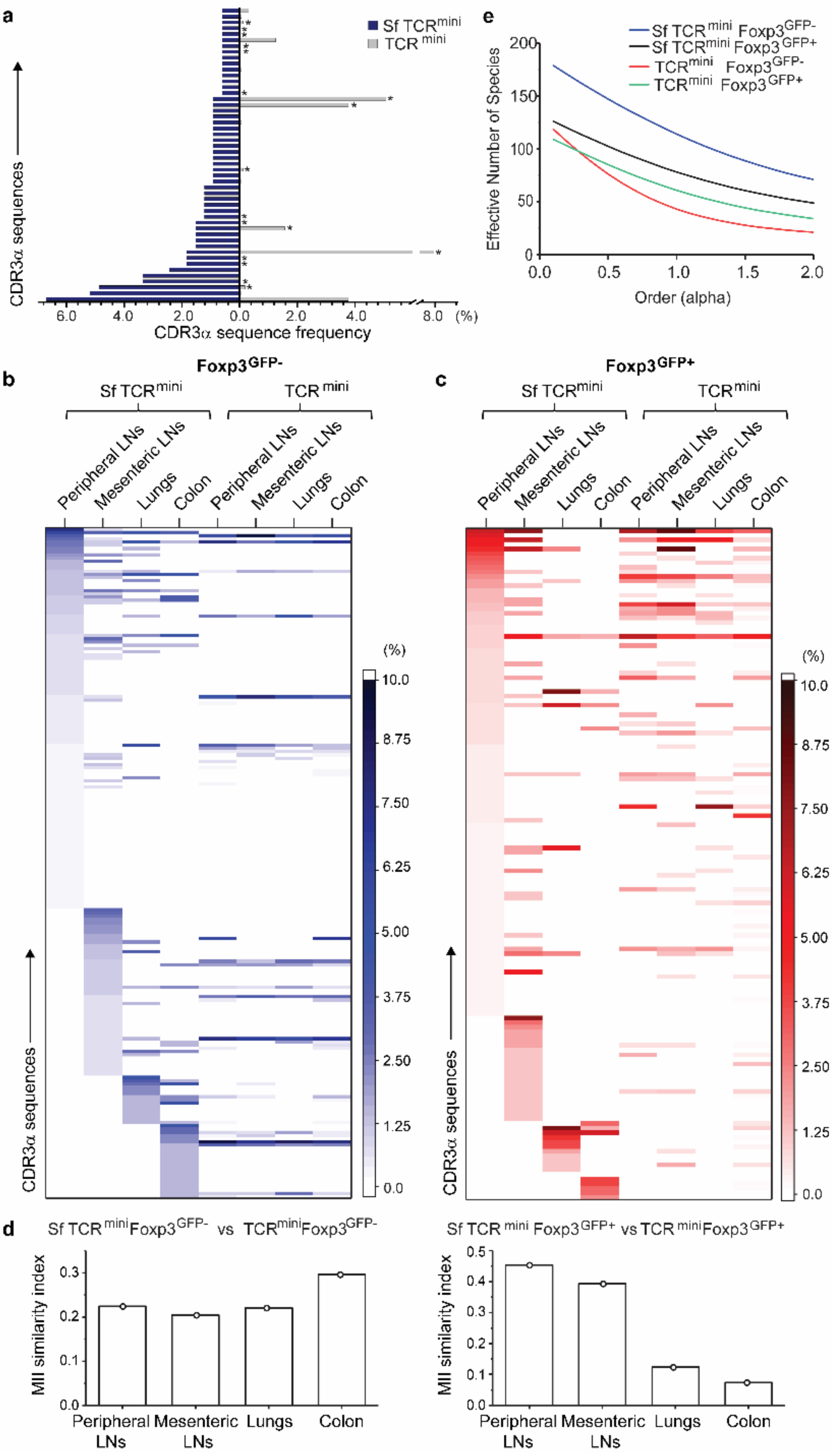

**Supplementary Figure 3. Overlap between TCRs on CD4$^+$ T cells from SfTCR$^{mini}$ and TCR$^{mini}$ mice.** This analysis was performed using single-cell RT-PCR followed by TCR$\alpha$ CDR3 sequencing. **a** Top fifty dominant TCRs expressed by Sf effectors (CD4$^+$CD44$^+$CD62L$^-$ Foxp3$^{GFP-}$) in LNs, and these TCRs frequencies on CD4$^+$Foxp3$^{GFP-}$ cells in the same organ in control TCR$^{mini}$ mice. Stars (*) mark TCRs also expressed by SfCD4$^+$ cell hybridomas that responded *ex vivo* to autologous DCs (shown in Figure 3). For CDR3$\alpha$ sequences see Supplementary Data 2**. b, c** Heatmaps depict frequencies of dominant TCRs for each CD4$^+$ subset from indicated organs (see Supplementary Data 3a and b for CDR3$\alpha$ sequences analyzed). Color shades reflect the relative frequency with which a given TCR was found in each organ. **d** Values of similarity indices (MII) for TCR repertoires from CD4$^+$Foxp3$^-$ and CD4$^+$Foxp3$^+$ populations. **e** Diversity index (REF) for TCR repertoires of CD4$^+$Foxp3$^{GFP-}$ (or Foxp3$^{GFP+}$) T cells from SfTCR$^{mini}$ and TCR$^{mini}$ mice.

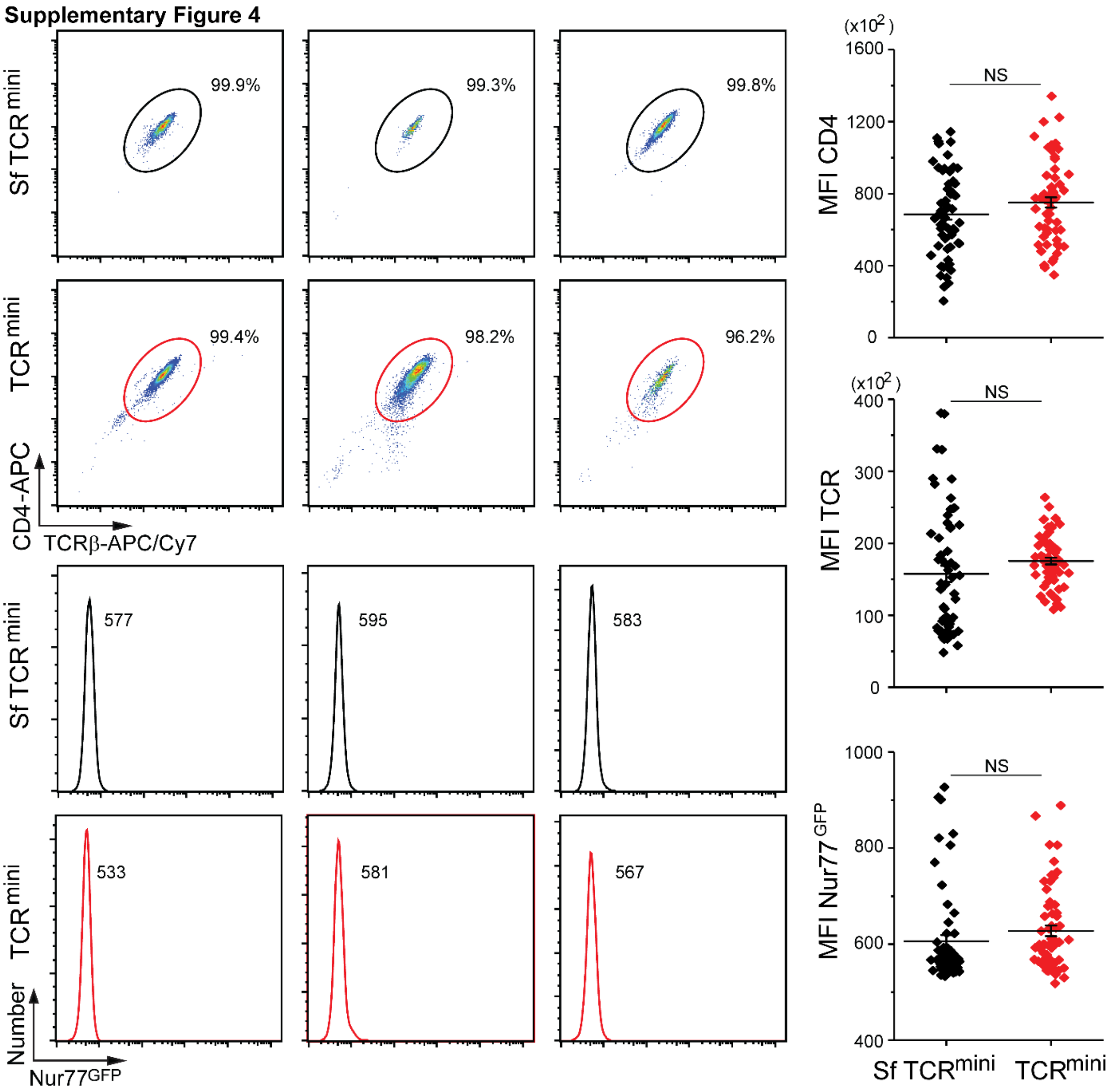


**Supplementary Figure 4. Expression of CD4, TCR and Nur77$^{GFP}$ on CD4$^{+}$Foxp3$^{-}$ hybridomas from SfTCR$^{mini}$ or TCR$^{mini}$ mice.** Dot on graphs represents one hybridoma (n=60). Unpaired Student t-test was applied, and statistical significance is indicated where appropriate (NS-not significant).

## Supplementary Figure 5

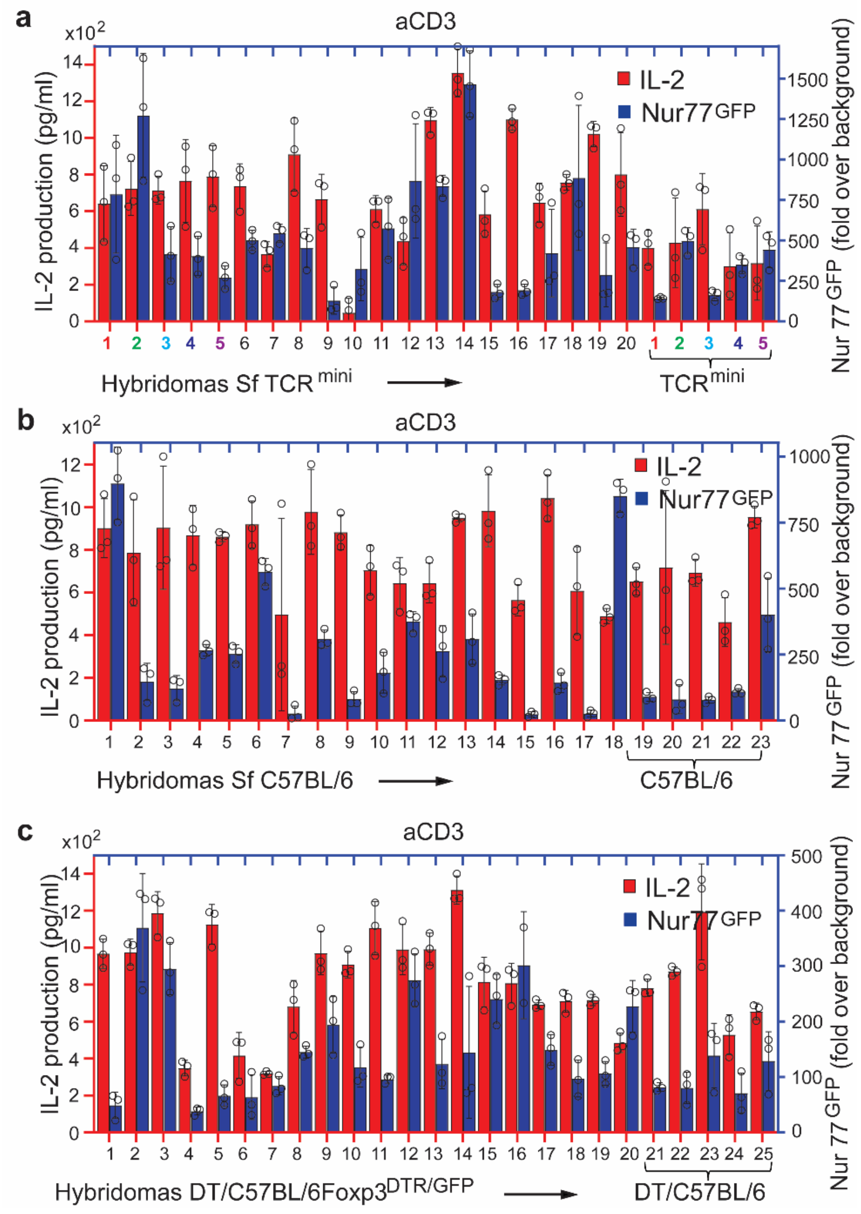


**Supplementary Figure 5. aCD3 activation of hybridomas: a** from SfCD4$^{+}$Foxp3$^{-}$ T cells, **b** from SfC57BL/6 or **c** DT/C57BL/6Foxp3$^{DTR/GFP}$ mice and their respective controls after overnight culture on aCD3 MoAb coated plates. For TCRα CDR3 sequences shown in Supplemental Figure 5a see Supplementary Data 4. Error bars are standard SD error across 3 replicas.

**Supplementary Figure 6**

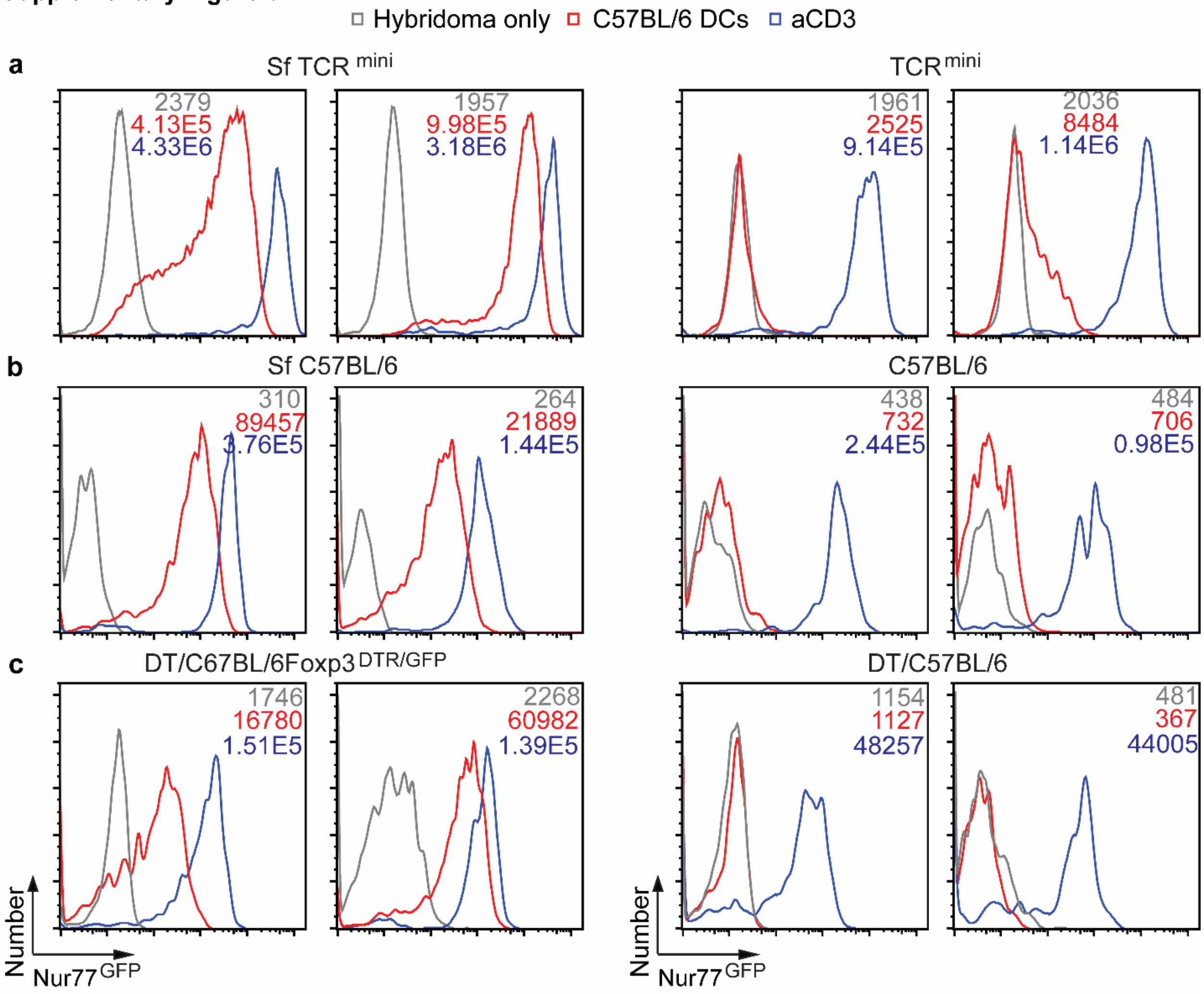


**Supplementary Figure 6. Nur77$^{GFP}$ on CD4$^{+}$Foxp3$^{-}$ hybridomas activated by C57BL/6 DCs or aCD3 MoAb.** Histograms depict responses of the first two hybridomas shown on each bar graph in Figure 3 or Supplementary Figure 5 from: **a** SfTCR$^{mini}$ and TCR$^{mini}$ mice, **b** SfC57BL/6 and C57BL/6 and **c** C57BL/6Foxp3$^{DTR/GFP}$ and control mice injected with the DT.

**Supplementary Figure 7**

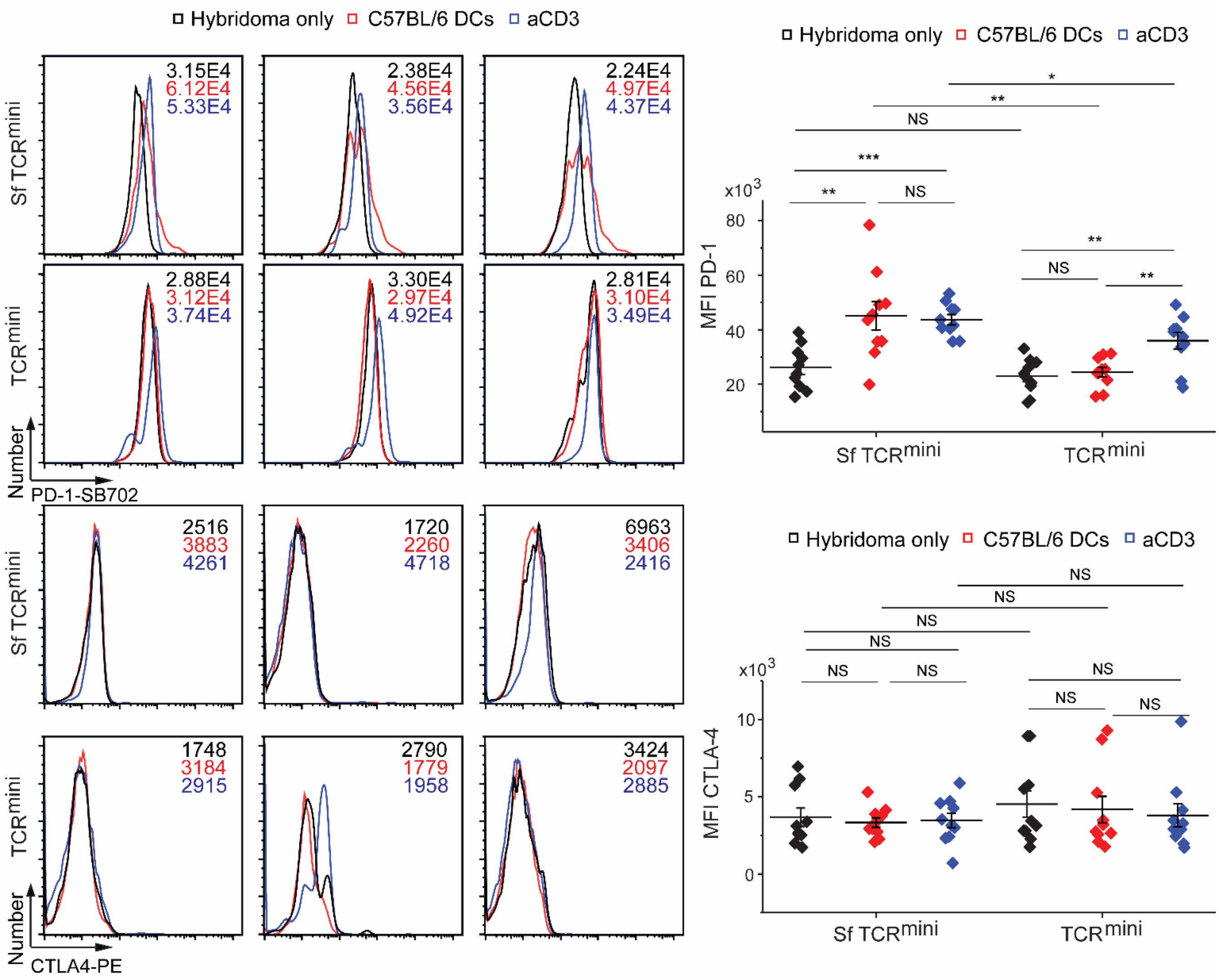


**Supplementary Figure 7. Expression of PD-1 and CTLA-4 on CD4$^+$Foxp3$^-$ hybridomas from SfTCR$^{mini}$ and TCR$^{mini}$ mice.** Dot on graphs represents one hybridoma (n=10). Student unpaired t-test was applied, and statistical significance (SD) is indicated where appropriate (* p<0.05, ** p<0.01, *** p<0.001, NS-not significant).

**Supplementary Figure 8**

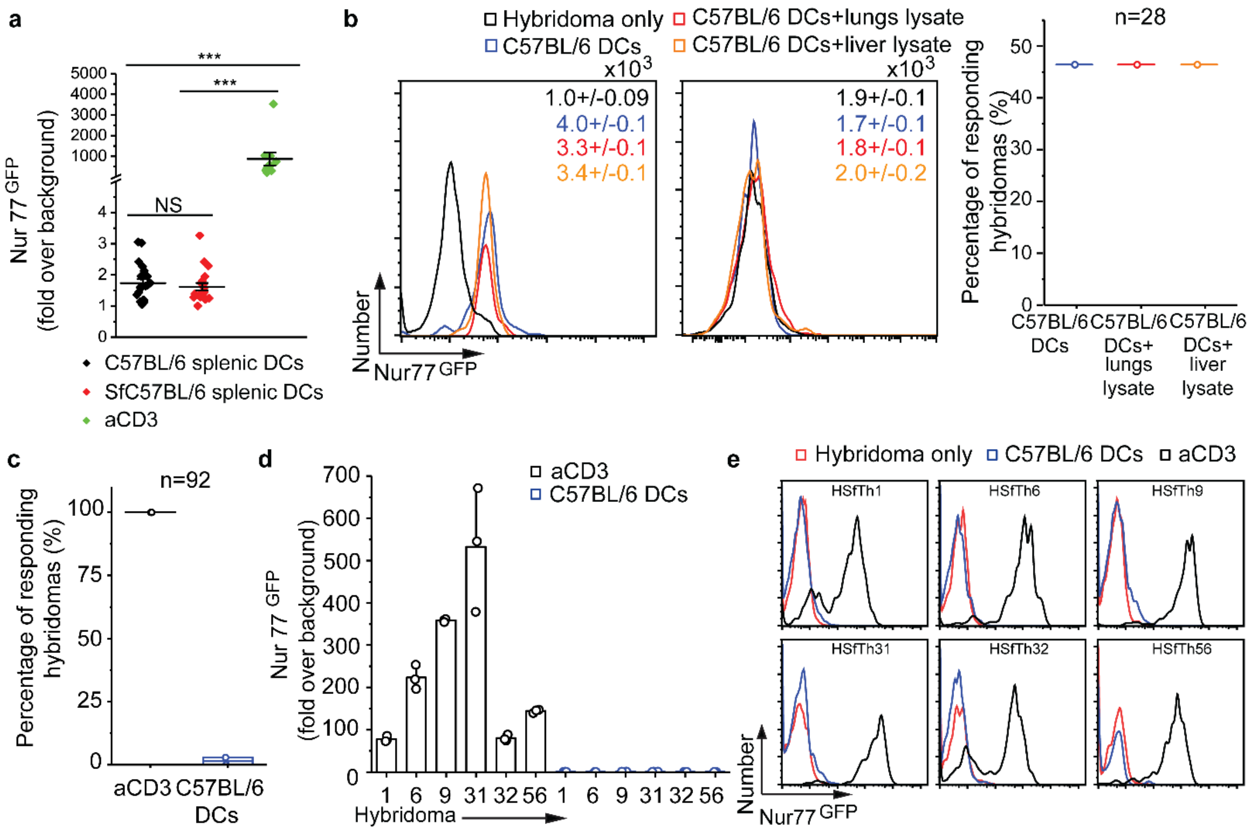


**Supplementary Figure 8. Hybridomas from SfCD4$^{+}$TCR$^{+}$ peripheral cells (but not thymocytes) are activated by autologous DCs. a** SfCD4Foxp3$^{-}$ hybridomas are similarly activated by splenic DCs from Sf and healthy mice. Representative responses of hybridomas (n=20) established from SfCD4$^{+}$Foxp3$^{GFP-}$ cells to DCs from SfTCR$^{mini}$ or C57BL/6 mice. Each symbol represents an individual hybridoma. Plate-bound aCD3 was used as a control. The average fold of Nur77$^{GFP}$ expression is shown (MFI GFP expression of hybridomas mixed with DCs divided by MFI GFP expression of hybridomas alone). Student t-test was applied, and statistical significance is indicated where appropriate (* p<0.05, ** p<0.01, *** p<0.001, NS-not significant). **b** Representative examples of hybridomas responses to autologous DCs preloaded overnight with cell lysates from indicated non-lymphoid organs. Graphs show a summary from 28 SfCD4$^{+}$Foxp3$^{GFP-}$ tested hybridomas. **c** Responses of hybridomas from Sf single positive thymocytes (CD4$^{+}$TCR$^{+}$Foxp3$^{GFP-}$) to plate-bound aCD3 MoAb or autologous DCs. Activation was measured by monitoring an increase in Nur77$^{\mathbf{GFP}}$ expression. **d** Graphs show fold change of MFI for Nur77$^{\mathbf{GFP}}$ reporter expression in thymocytes-derived hybridomas after these cells activation with indicated stimulants. Histograms show representative results from one of three independent experiments. Error bars depict SD error across 3 replicas. **e** Hybridomas established from SfCD4$^{+}$Foxp3$^{GFP-}$ thymocytes respond to stimulation by immobilized aCD3 but not to autologous DCs. Responses were detected using Nur77$^{GFP}$ reporter.

**Supplementary Figure 9**

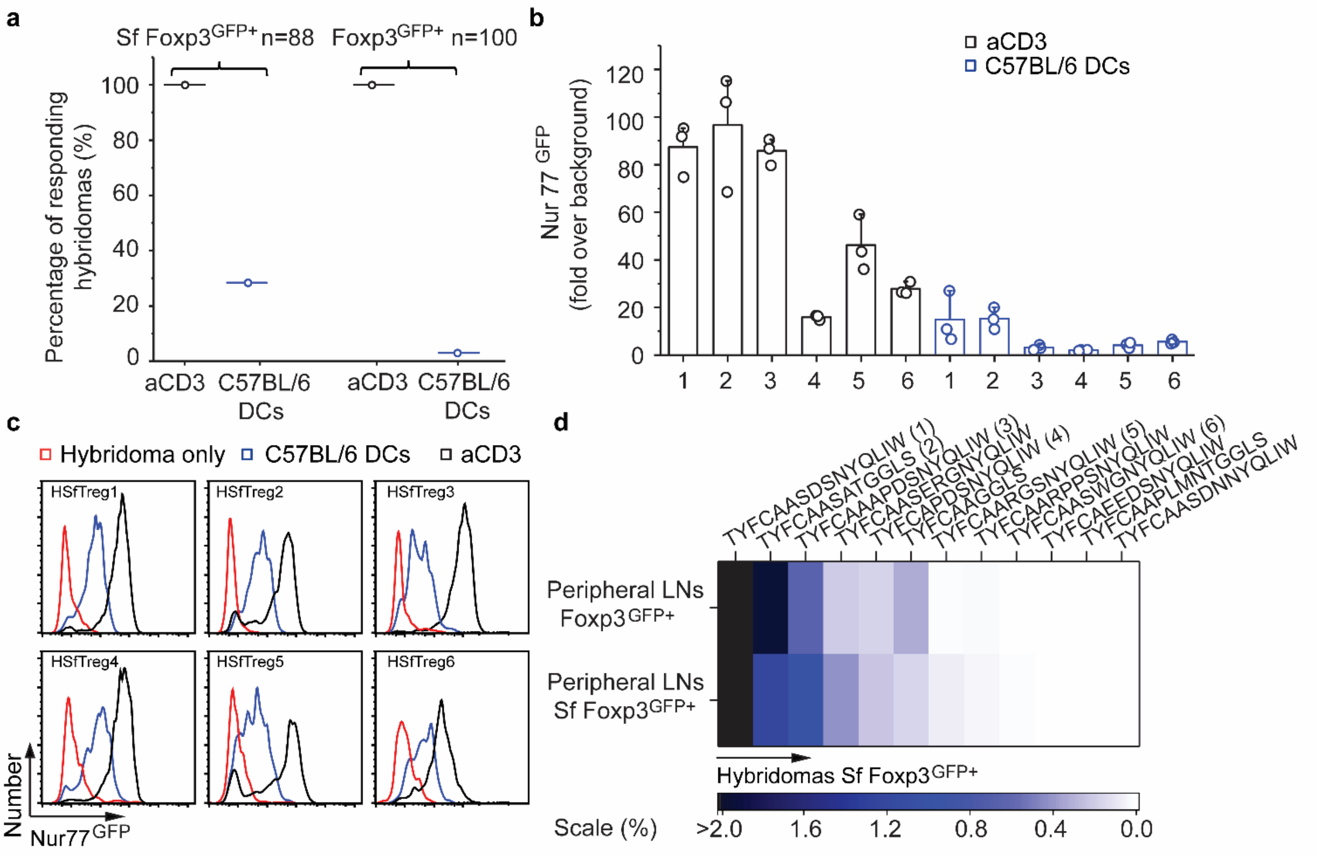


**Supplementary Figure 9. Hybridomas from disabled SfCD4$^{+}$Foxp3$^{GFP+}$ respond to autologous DCs. a** Percentage of hybridomas established from CD4Foxp3$^{GFP+}$ cells from SfTCR$^{mini}$ or TCR$^{mini}$ mice that responded to stimulation with aCD3 MoAb or autologous APCs. **b, c** Graphs show representative responses of six (total of 88) hybridomas produced from SfCD4$^{+}$Foxp3$^{GFP+}$ cells to aCD3 and autologous DCs, measured by Nur77$^{GFP}$ upregulation. Error bars are standard SD error across 3 replicas. Histograms show representative results from one of three independent experiments. **d** Sequences of TCRα CDR3 region and these TCRs frequencies on CD4$^{+}$Foxp3$^{GFP+}$ cells in peripheral lymph nodes in both investigated strains of TCR$^{mini}$ mice. Quantitative estimates of TCRs abundance on CD4$^{+}$Foxp3$^{GFP+}$ cells were done based on HTS of TCRs from these subsets. Error bars are standard error across 3 replicas.

**Supplementary Figure 10**

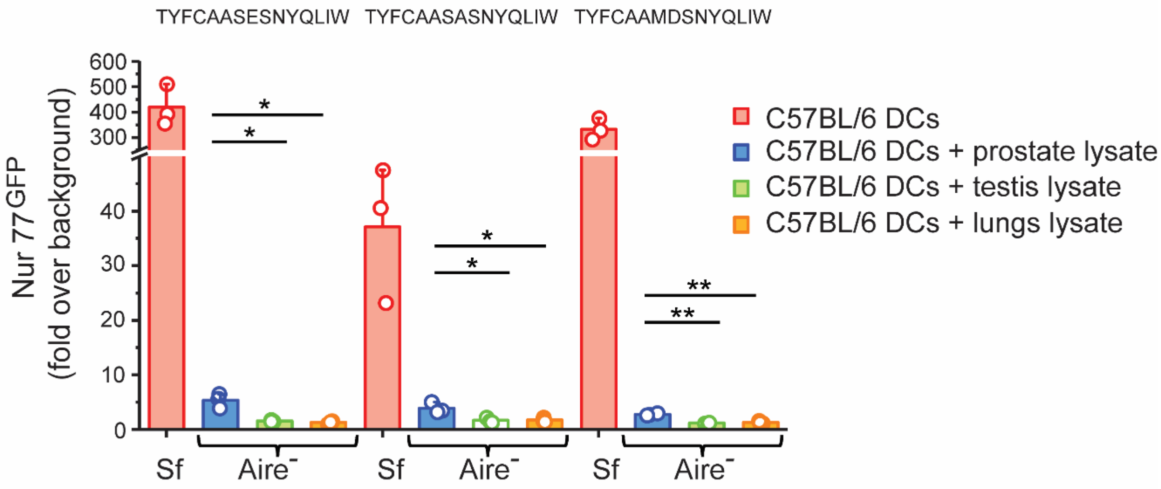


**Supplementary Figure 10. Tregs deficits in SfTCR$^{mini}$ and TCR$^{mini}$Aire$^{-}$ differently impact autoresponses of CD4$^{+}$Foxp3$^{-}$ cells.** Graphs show activation of hybridomas from SfCD4$^{+}$Foxp3$^{-}$ cells by C57BL/6 DCs, whereas hybridomas from Aire$^{-}$CD4$^{+}$Foxp3$^{-}$ are activated by exclusively prostate autoantigens. Three pairs of hybridomas expressing mutual TCRs are shown. Student paired t-test was applied, and statistical significance is indicated where appropriate (* p<0.05, ** p<0.01, *** p<0.001). Error bars are SD across 3 replicas.

**Supplementary Figure 11**

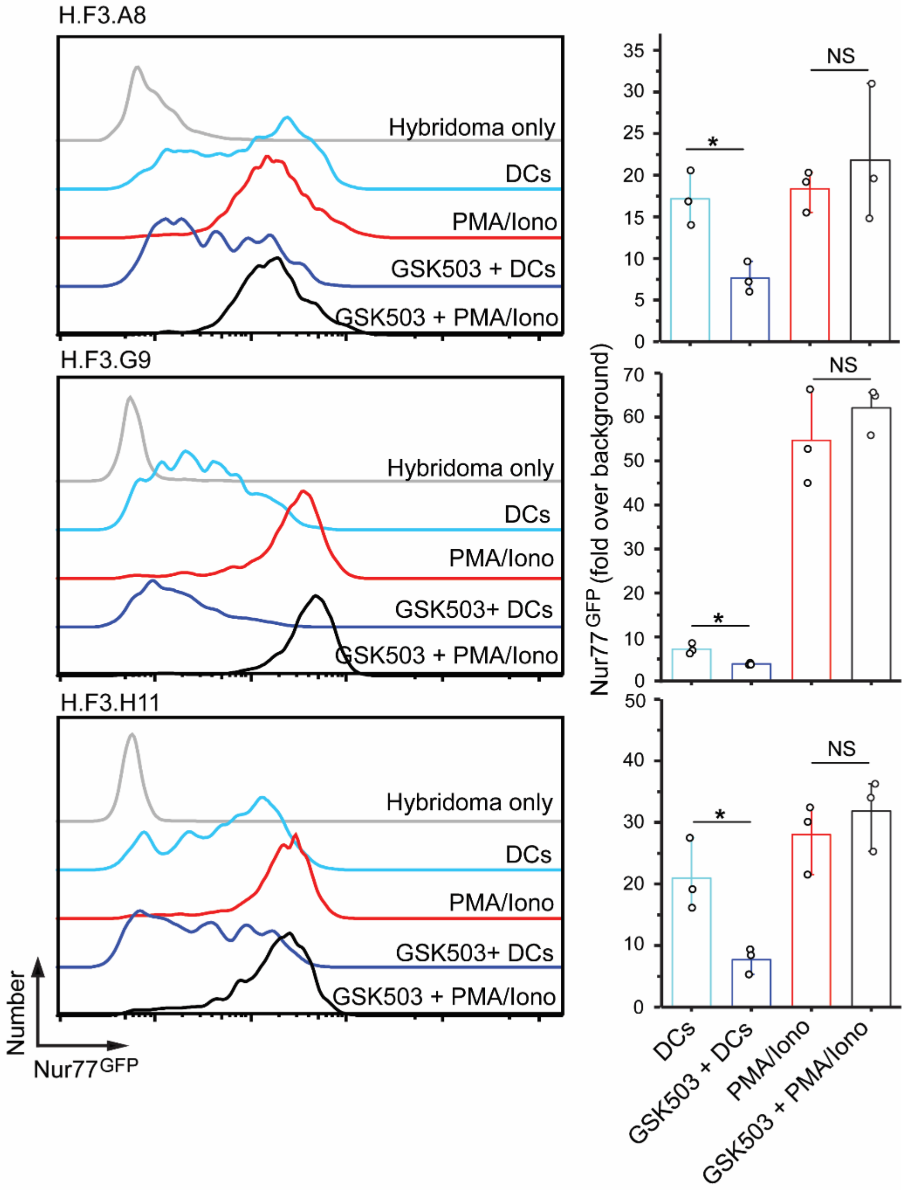


**Supplementary Figure 11. Pharmacological inhibitor of Ezh2 ameliorates auto response of CD4$^{+}$Foxp3$^{-}$ hybridomas.** Histograms depict changes in Nur77$^{GFP}$ expression to the indicated stimulant in 3 representative hybridomas (of total 10 tested). Bar graphs show a summary of changes in Nur77$^{GFP}$ reporter expression for each tested hybridoma. Hybridomas represent CD4$^{+}$Foxp3$^{-}$ cells from C57BL/6Foxp3$^{DTR/GFP}$ mice after Tregs ablation with DT. Student paired t-test was applied, and statistical significance is indicated where appropriate (* p<0.05, ** p<0.01, *** p<0.001, NS- not significant). Error bars are SD across 3 replicas.

**Supplementary Figure 12**

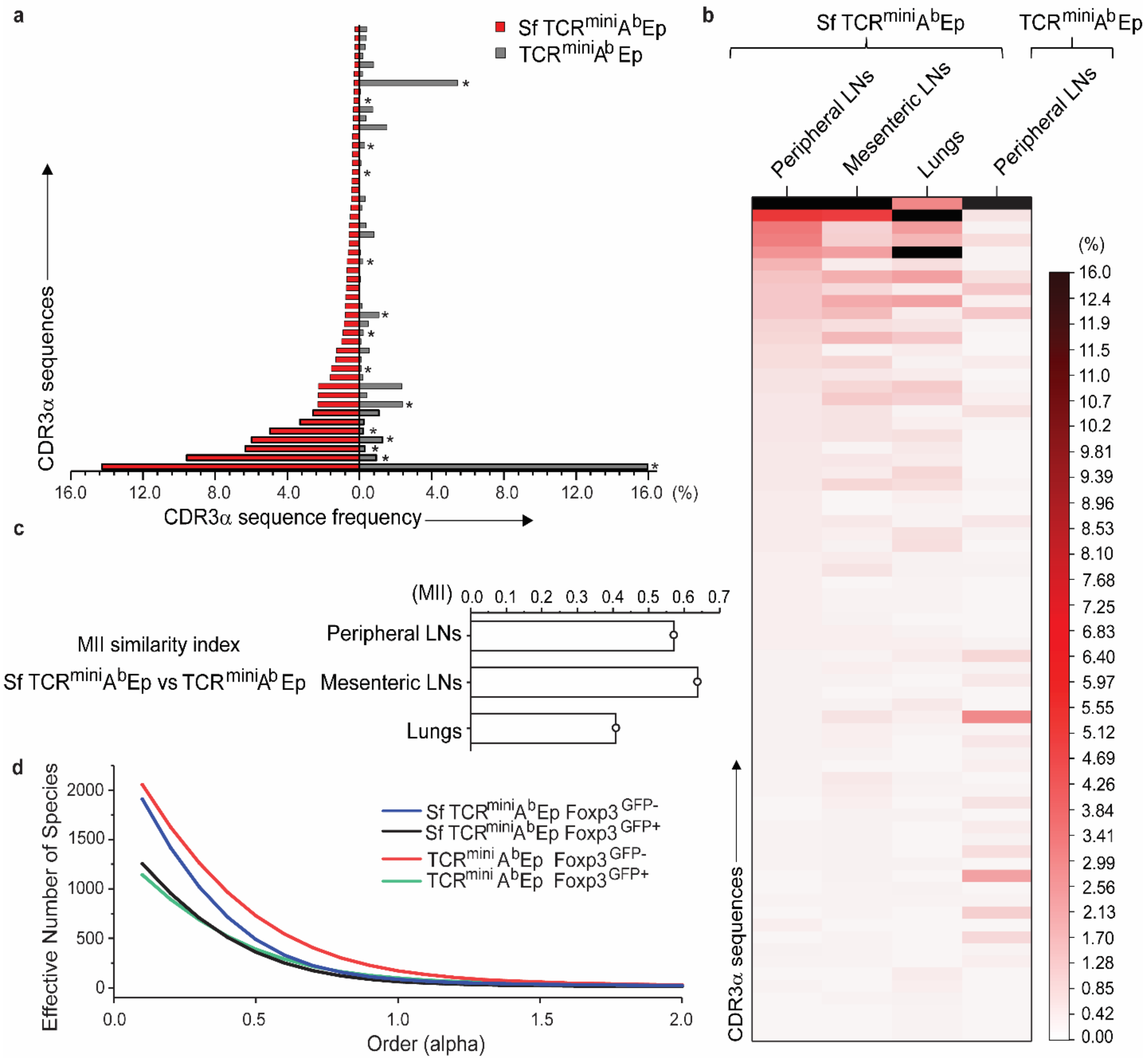


**Supplementary Figure 12. Autoreactive TCRs from CD4$^+$Foxp3$^{GFP-}$ clones in SfTCR$^{mini}$A$^b$Ep mice are often expressed by CD4$^+$Foxp3$^{GFP-}$ cells from healthy TCR$^{mini}$A$^b$Ep mice. a** 50 dominant TCRs expressed by CD4$^+$Foxp3$^{GFP-}$ cells from SfTCR$^{mini}$A$^b$Ep mice (red bars) and their frequencies on peripheral CD4$^+$Foxp3$^{GFP-}$ cells in TCR$^{mini}$A$^b$Ep mice (grey bars). Asterisks denote TCRs also expressed on autoreactive hybridomas (for sequences see Supplementary Data 7). **b** Frequencies of autoreactive TCRs on CD4$^+$Foxp3$^{GFP-}$ effectors from organs of SfTCR$^{min}$A$^b$Ep $^{mice}$ and these TCRs frequencies on peripheral CD4Foxp3$^{GFP-}$ cells from TCR$^{mini}$A$^b$Ep mice (see Supplementary Data 8 for sequences). **c** Similarity indices (MII) for indicated TCR repertoires of CD4$^+$Foxp3$^{GFP-}$ cells. **d** Relative diversities of TCRs on CD4$^+$ subsets from indicated strains. TCRs data were combined from 3 individually sequenced mice.

**Supplementary Figure 13**

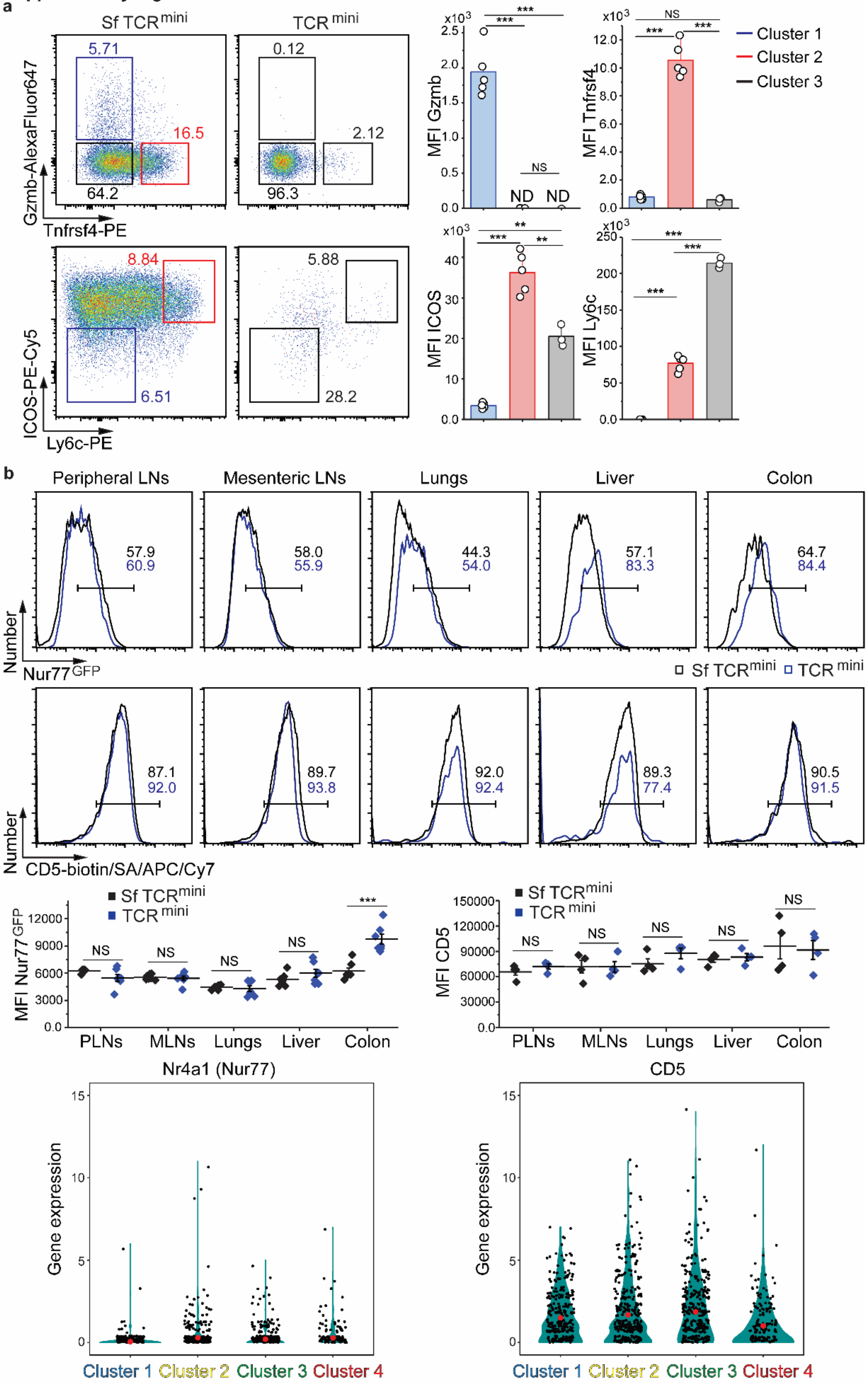

**Supplementary Figure 13. Validation of discriminating and non-discriminating expression of selected molecules by activated $CD4^{+}CD44^{+}CD62L^{+}$ from $SfTCR^{mini}$ and $TCR^{mini}$ mice. a** Expression of Gzmb vs Tnfrsf4 and ICOS vs Ly6c on activated $CD4^{+}$ cells from both studied here strains of $TCR^{mini}$ mice using flow cytometry. Also see Figure 7b, c. Error bars depict SD error across 3 replicas. **b** Expression of $Nur77^{GFP}$ reporter and $CD5^{+}$ by a subset of $CD4^{+}CD44^{+}CD62L^{-}$ T cells from $SfTCR^{mini}$ and $TCR^{mini}$ mice using flow cytometry. Graphs show % of cells expressing indicated molecules. Each symbol represents one analyzed sample. Violin plots show expression of Nur77 and CD5 single cells by scRNAseq. Student unpaired t-test was applied, and statistical significance is indicated where appropriate (* $p<0.05$, ** $p<0.01$, *** $p<0.001$, NS-not significant).

## Supplementary Figure 14

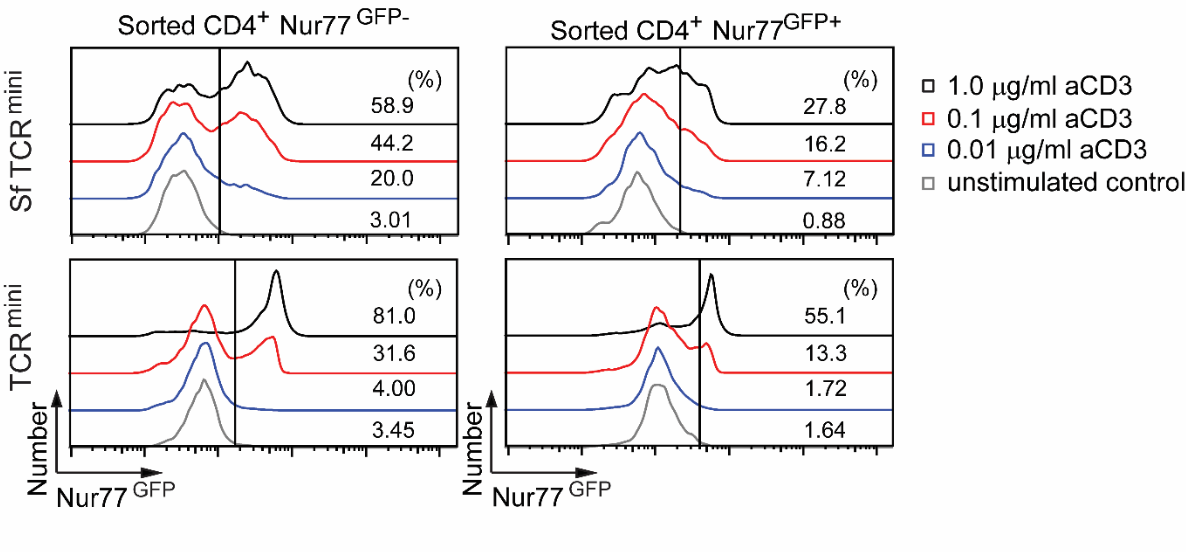


**Supplementary Figure 14. *Ex vivo* SfCD4⁺ cells have a higher sensitivity to a weak TCR activation as compared to their counterparts from control mice.** Freshly isolated peripheral CD4⁺ cells were activated overnight with various concentrations of plate-bound aCD3 MoAb, and the expression of Nur77$^{GFP}$ reporter was measured by FACS. Histograms show representative *ex vivo* expression of indicated GFP reporter.

Supplementary Figure 15

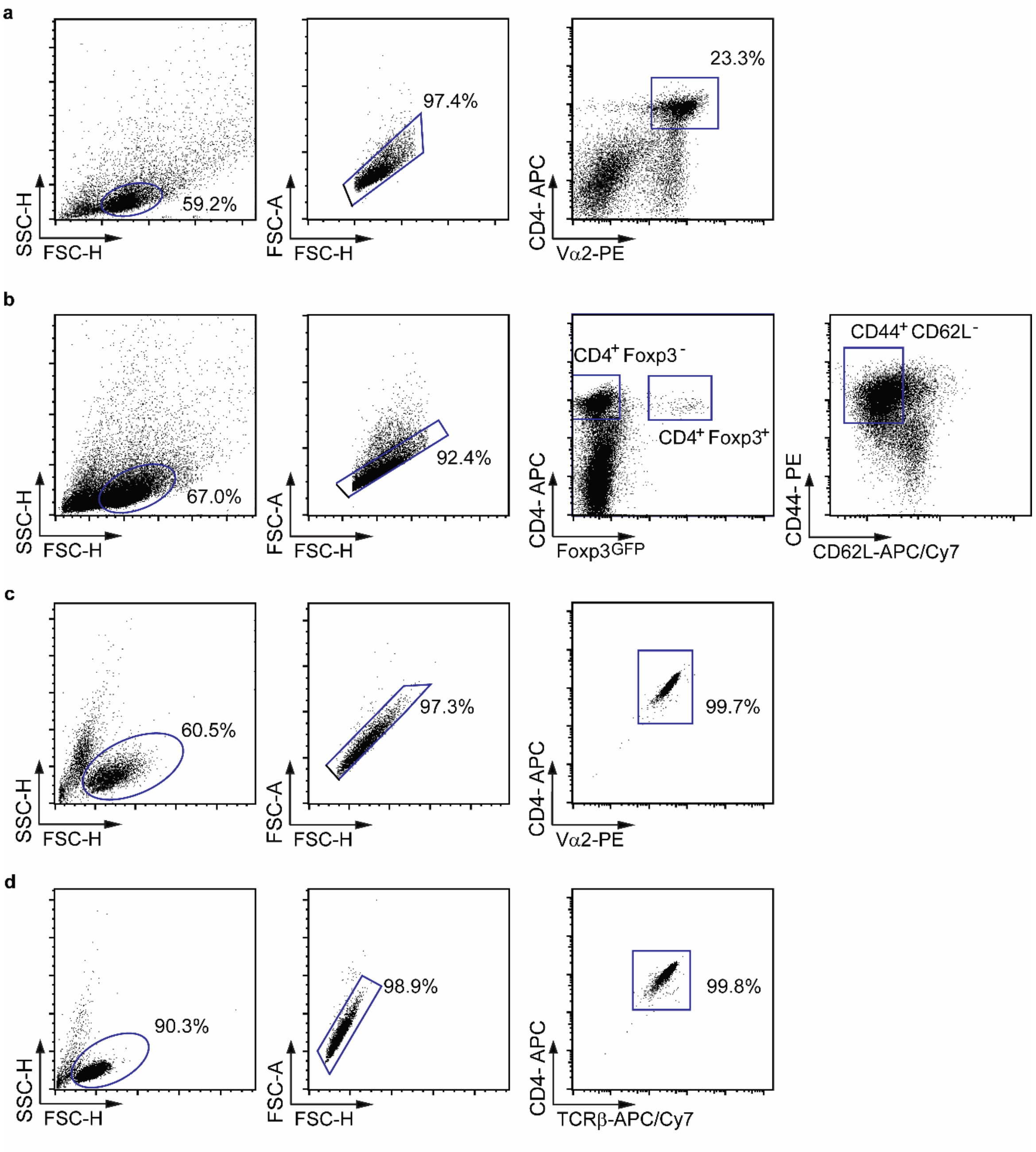


**Supplementary Figure 15. Gating strategy.**

**a** for Figures 1,4, 6 and Supplementary Figure. 1 and 14.

**b** for Figures 2, 7, Supplementary Figure 3 and 12.

**c** for Figures 3, 5, Supplementary Figure 4, 5a, 6a, 7, 8, 9, 10 and 13.

**d** for Supplementary Figure. 5b, c, 6b, c and 11.

**Supplementary Table 1**

| antibody | clone | conjugate | concentration used in the study [mg/ml] | supplier | catalog number |
|---|---|---|---|---|---|
| CD3 | 145-2C11 | unconjugated | variable (0.0001-5) | BioLegend | 100331 |
| CD4 | GK1.5 | BV510 | 0.8 | BD | 743155 |
| CD4 | GK1.5 | APC | 0.2 | BioLegend | 100412 |
| CD4 | GK1.5 | biotin | 0.4 | BioLegend | 100404 |
| CD5 | 53-7.3 | biotin | 0.2 | BioLegend | 100604 |
| CD8a | 53-6.7 | biotin | 0.4 | BioLegend | 100704 |
| CD28 | 37.51 | unconjugated | 1 or 5 | BioLegend | 102112 |
| CD44 | IM7 | eFluor450 | 0.4 | eBioscience | 48-0441-80 |
| CD44 | IM7 | PE | 0.8 | BioLegend | 103008 |
| CD62L | MEL14 | APC-Cy7 | 0.4 | BioLegend | 104428 |
| CD71 | RI7217 | PE-Cy7 | 0.8 | BioLegend | 113812 |
| CD134 (OX40) | OX-86 | PE | 0.8 | BioLegend | 119410 |
| CD278 (ICOS) | 15F9 | PE-Cy5 | 0.8 | BioLegend | 107708 |
| Granzyme B | GB11 | Alexa Fluor 647 | 0.8 | BioLegend | 515405 |
| Ly6C | HK1.4 | PE | 0.2 | BioLegend | 128007 |
| CD279 (PD-1) | J43 | SB702 | 0.4 | eBioscience | 67-9985-80 |
| CD152(CTLA-4) | UC10-4f10 | PE | 0.1 | Pharmingen | 553720 |
| streptavidin | streptavidin | PE-Cy7 | 0.4 | BioLegend | 405206 |
| streptavidin | streptavidin | APC-Cy7 | 0.2 | BioLegend | 405208 |
| streptavidin | streptavidin | PE-Cy5 | 0.2 | BioLegend | 405205 |
| V$\alpha$2 | B20.1 | PE | 0.4 | BD | 553289 |
| V$\beta$14 | 14-2 | biotin | 0.4 | BD | 553257 |
| TCR$\beta$ | H57-597 | APC-Cy7 | 0.4 | BioLegend | 109220 |

**Supplementary Table 2**

| Chemicals, Peptides, and Recombinant Proteins | Source | Identifier |
|---|---|---|
| Recombinant Murine GM-CSF | Peprotech | 315-03-50UG |
| Recombinant Murine IL-2 | Peprotech | 212-12-5UG |
| Lymphocyte Separation Medium | Corning | 25-072-CV |
| Collagenase D | Roche | 11088882001 |
| DNase I | Roche | 10104159001 |
| Neomycin trisulfate salt hydrate | Sigma | N6386-100G |
| SuperScript III First-Strand Synthesis System | Invitrogen | 18080051 |
| AccuPrime Taq DNA Polymerase | Invitrogen | 12346-086 |
| MMLV reverse transcriptase | Promega | M1701 |
| Perfect Taq Polymerase | 5 PRIME | 2200070 |

| | | |
|---|---|---|
| GSK 503 | Cayman Chemicals | 18531 |
| Diphtheria toxin | Sigma | 322326 |
| PMA | Sigma | P8139-5mg |
| Ionomycin | Sigma | I0634-1mg |

**Supplementary Table 3**

| Critical Commercial Assays | Source | Identifier |
|---|---|---|
| RNeasy Mini Kit | Qiagen | 74106 |
| NucleoFast® 96 PCR Plates | Macherey-Nagel | 743100.50 |
| Fixation/Permeabilization solution | Invitrogene | 50-112-9060 |
| 10x Permeabilization buffer | eBiosicience | 00-8333-56 |
| Agilent Bioanalyzer High SensitivityDNA Kit | Agilent | 5067-4626 |
| Chromium™ i7 Multiplex Kit, 96 rxns | 10x Genomics | 120262 |
| Chromium™ Single Cell 3'/5' Library Construction Kit, 16 rxns | 10x Genomics | 1000020 |
| Chromium™ Single Cell 5' Library & Gel Bead Kit, 16 rxns | 10x Genomics | 1000006 |

**Supplementary Table 4**

| Experimental Models: Organisms/Strains | Source | Identifier |
|---|---|---|
| Mouse: SfC57BL/6 | Jackson laboratory | 004088 |
| Mouse: TCR$\alpha^{-}$ | Jackson laboratory | 002116 |
| Mouse: C57BL/6Foxp3$^{GFP}$ | Jackson laboratory | 023800 |
| Mouse: TCR$^{mini}$ | (Pacholczyk et al., 2006) | |
| Mouse: TCR$^{mini}$A$^{b}$Ep, TCR$^{mini}$A$^{b}$Ep63K | (Wojciech et al., 2014) | |
| Mouse: SfTCR$^{mini}$ | This paper | |
| Mouse TCR$^{mini}$Aire$^{-}$ | (Danielly et al, 2010) | |
| Mouse: SfTCR$^{mini}$A$^{b}$Ep | This paper | |
| Mouse: C57BL/6Nur77$^{GFP}$ | Jackson laboratory | 016617 |
| Mouse: C57BL/6 Foxp3$^{DTR/GFP}$ | Jackson laboratory | 016958 |
| Mouse: H2M$^{-}$Ii$^{-}$ | (Kraj et al., 2001) | |
| Cell line: HT-2 | (Pacholczyk et al., 2007) | |
| Cell line: BWNur77$^{GFP}$ | (manuscript in preparation) | |
| Cell line: T cell hybridomas | This paper | |

**Supplementary Table 5**

| Oligonucleotide sequences | Source |
|---|---|
| ACACTCTTTCCCTACACGACGCTCTTCCGATCTACAGACTCTCAGCCTGGAGACTCAGCT | This paper |
| GTGACTGGAGTTCAGACGTGTGCTCTTCCGATCTTTAACTGGTACACAGCAG | This paper |
| CAAGCAGAAGACGGCATACGAGATXXXXXXXXGTGACTGGAGTTCAGACGTGTGCTCTTCCGATC | This paper |

| | |
|---|---|
| AATGATACGGCGACCACCGAGATCTACACXXXXXXXXACACTCTTTCCCTACACGACGCTCTTCCGATCT | This paper |

**Supplementary Table 6**

| Software and Algorithms | Source | Identifier |
|---|---|---|
| OriginPro v2017 | OriginLab | https://www.originlab.com |
| FlowJo v10 | FlowJo LLC | https://www.flowjo.com/ |
| Python-based custom TCR extraction tool | (Szurek et al., 2015) | |
| Custom made TCR database | (Cebula et al., 2013) | |
| Cell Ranger v2.0 | 10X Genomics | https://support.10xgenomics.com/single-cell-gene-expression/software/pipelines/latest/what-is-cell-ranger |
| CLoupe and VLoupe v1.05 | 10X Genomics | https://support.10xgenomics.com/single-cell-gene-expression/software/downloads/latest |

## Contact for Reagent and Resource Sharing

Further information and requests for reagents should be directed to and will be fulfilled by the Corresponding Author: Leszek Ignatowicz lignatowicz@gsu.edu
