# Supplementary material for: Dormant pathogenic CD4+ T cells are prevalent in the peripheral repertoire of healthy mice
Source: Nat Commun. 2019 Oct 25;10:4882. doi: 10.1038/s41467-019-12820-3 (PMC6814812; doi:10.1038/s41467-019-12820-3)
Supplement: Supplementary file 3 — Supplementary Data [file 41467_2019_12820_MOESM3_ESM.docx]

**Supplementary Data:**

**Supplementary Data 1. Amino acid sequences of TCRα CDR3 regions shown in** **Figure 2a and 2b.**

| **1a**  Foxp3^GFP-^ | | **1b** Foxp3^GFP+^ | |
| --- | --- | --- | --- |
| 1 | TYFCAARDSNYQLIW | 1 | TYFCAASDSNYQLIW |
| 2 | TYFCAASESNYQLIW | 2 | TYFCAARDSNYQLIW |
| 3 | TYFCAASDSNYQLIW | 3 | TYFCAAEDGNYQLIW |
| 4 | TYFCAAMDSNYQLIW | 4 | TYFCAATMDSNYQLIW |
| 5 | TYFCAANSNYQLIW | 5 | TYFCAATSNTGGLS |
| 6 | TYFCAASLNTGGLS | 6 | TYFCAASPYSNYQLIW |
| 7 | TYFCAASPMNTGGLS | 7 | TYFCAATSSNYQLIW |
| 8 | TYFCAASRGGLS | 8 | TYFCAARGWSNYQLIW |
| 9 | TYFCAAKDSNYQLIW | 9 | TYFCAASPMNTGGLS |
| 10 | TYFCAAKGSNYQLIW | 10 | TYFCAASGDTGGLS |
| 11 | TYFCAASAHSNYQLIW | 11 | TYFCAASAGTGGLS |
| 12 | TYFCAANTGGLS | 12 | TYFCAASPENTGGLS |
| 13 | TYFCAASNTGGLS | 13 | TYFCAANDSNYQLIW |
| 14 | TYFCAASPDSNYQLIW | 14 | TYFCAASGGNYQLIW |
| 15 | TYFCAASASNYQLIW | 15 | TYFCAADTRNYQLIW |
| 16 | TYFCAASARSNYQLIW | 16 | TYFCAARPPQGNYQLIW |
| 17 | TYFCAARESNYQLIW | 17 | TYFCAASAQGNYQLIW |
| 18 | TYFCAASEGNYQLIW | 18 | TYFCAAKAGGNYQLIW |
| 19 | TYFCAASGNTGGLS | 19 | TYFCAARNSNYQLIW |
| 20 | TYFCAAHNSNYQLIW | 20 | TYFCAASGTGGLS |
| 21 | TYFCAASDTGGLS | 21 | TYFCAVNTGGLS |
| 22 | TYFCAASGTGGLS | 22 | TYFCAATGGNYQLIW |
| 23 | TYFCAASMDSNYQLIW | 23 | TYFCAASESNYQLIW |
| 24 | TYFCAAIDDYQLIW | 24 | TYFCAQMDSNYQLIW |
| 25 | TYFCAAINYQLIW | 25 | TYFCAALWDTGGLS |
| 26 | TYFCAAIRHSNYQLIW | 26 | TYFCAAYSHSNYQLIW |
| 27 | TYFCAAIYDQLIW | 27 | TYFCAASAESNYQLIW |
| 28 | TYFCAAKDRGGLS | 28 | TYFCAASAGGSNYQLIW |
| 29 | TYFCAARAHSNYQLIW | 29 | TYFCAAIDDYQLIW |
| 30 | TYFCAARASNYQLIW | 30 | TYFCAASEKGNYQLIW |
| 31 | TYFCAARMDSNYQLIW | 31 | TYFCAASEPNYQLIW |
| 32 | TYFCAASANSNYQLIW | 32 | TYFCAASFVDSNYQLIW |
| 33 | TYFCAASAQGGGLS | 33 | TYFCAASGASNYQLIW |
| 34 | TYFCAASAVGYQLIW | 34 | TYFCAASGDSNYQLIW |
| 35 | TYFCAASAVSNYQLIW | 35 | TYFCAASEDQLIW |
| 36 | TYFCAASAVWTGGLS | 36 | TYFCAASARSNYQLIW |
| 37 | TYFCAASDRMGNYQLIW | 37 | TYFCAASASNYQLIW |
| 38 | TYFCAASETGGLS | 38 | TYFCAASAVSNYQLIW |
| 39 | TYFCAASFVDSNYQLIW | 39 | TYFCAASGNSNYQLIW |
| 40 | TYFCAASGANTGGLS | 40 | TYFCAAMNSNYQLIW |
| 41 | TYFCAASGDQLIW | 41 | TYFCAAMDSNYQLIW |
| 42 | TYFCAASGGLS | 42 | TYFCAAAPDSNYQLIW |
| 43 | TYFCAASPENTGGLS | 43 | TYFCAADAWSNYQLIW |
| 44 | TYFCAASWNDYQLIW | 44 | TYFCAAEARNYQLIW |
| 45 | TYFCAATDDYQLIW | 45 | TYFCAASDDNYQLIW |
| 46 | TYFCAATGGNYQLIW | 46 | TYFCAASDGYQLIW |
| 47 | TYFCAAWRHSNYQLIW | 47 | TYFCAAETGGLS |
| 48 | TYFCAAWSNYQLIW | 48 | TYFCAAREGSNYQLIW |
| 49 | TYFCAAWTHSNYQLIW | 49 | TYFCAARAPFNYQLIW |
| 50 | TYFCANEDSNYQLIW | 50 | TYFCAASAKRVSNYQLIW |
| 51 | TYFCAQLNTGGLS | 51 | TYFCAASANSNYQLIW |
| 52 | TYFCGEDSNYQLIW | 52 | TYFCAASAGSTGGLS |
| 53 | TYFCAADNRNYQLIW | 53 | TYFCAAMEGSNYQLIW |
| 54 | TYFCAAFTDSNYQLIW | 54 | TYFCAAKYGGNYQLIW |
| 55 | TYFCAASHGTGGLS | 55 | TYFCANEDSNYQLIW |
| 56 | TYFCAASDVRGDSNYQLIW | 56 | TYFCAVIRHSNYQLIW |
| 57 | TYFCAASEWEYQLIW | 57 | TYFCAYLDSNYQLIW |
| 58 | TYFCAASEDTGGLS | 58 | TYFCAASTNNYQLIW |
| 59 | TYFCAANDGGGLS | 59 | TYFCAASPGFRQLIW |
| 60 | TYFCAASDSRGDSNYQLIW | 60 | TYFCKIGSNYQLIW |
| 61 | TYFCAASAAHQLIW | 61 | TYFCAAGEGNYQLIW |
| 62 | TYFCAATPPKGNYQLIW | 62 | TYFCAASNSNYQLIW |
| 63 | TYFCAAITGGLS | 63 | TYFCAASRAGSNYQLIW |
| 64 | TYFCAASDGDSNYQLIW | 64 | TYFCGSEDSNYQLIW |
| 65 | TYFCAASAQGNYQLIW | 65 | TYFCAVLGQGGNYQLIW |
| 66 | TYFCAASTNNYQLIW | 66 | TYFCAVDSNYQLIW |
| 67 | TYFCAASMGSNYQLIW | 67 | TYFCAARWTGGLS |
| 68 | TYFCAASDVKGDSNYQLIW | 68 | TYFCAASASSNYQLIW |
| 69 | TYFCAAIKHSNYQLIW | 69 | TYFCAASGAGSNYQLIW |
| 70 | TYFCAAEDGNYQLIW | 70 | TYFCAASGAYSNYQLIW |
| 71 | TYFCAASSHSNYQLIW | 71 | TYFCAASNYQLIW |
| 72 | TYFCAADTRNYQLIW | 72 | TYFCAASRGSNYQLIW |
| 73 | TYFCAASGWSNYQLIW | 73 | TYFCAASAAHQLIW |
| 74 | TYFCAASDGYQLIW | 74 | TYFCADPLDSNYQLIW |
| 75 | TYFCADLGSNYQLIW | 75 | TYFCAAIGDQLIW |
| 76 | TYFCAASFAGTGGLS | 76 | TYFCAAKWWGNYQLIW |
| 77 | TYFCAASDGGLS | 77 | TYFCAATDDYQLIW |
| 78 | TYFCAAKGWTGGLS | 78 | TYFCAASVDSNYQLIW |
| 79 | TYFCAALRHSNYQLIW | 79 | TYFCAQLNTGGLS |
| 80 | TYFCAADGSNYQLIW | 80 | TYFCGEDSNYQLIW |
| 81 | TYFCAASWSATGGLS | 81 | TYFCAASWTGGLS |
| 82 | TYFCAASGDAGGLS | 82 | TYFCAARDGGSNYQLIW |
| 83 | TYFCAAISRGSNYQLIW | 83 | TYFCAAMNTGGLS |
| 84 | TYFCAASGWAGGLS | 84 | TYFCAAQLGGNYQLIW |
| 85 | TYFCAASEDQLIW | 85 | TYFCAAKQGGNYQLIW |
| 86 | TYFCAASSSNYQLIW | 86 | TYFCAASATGGLS |
| 87 | TYFCAAQDSNYQLIW | 87 | TYFCAAFNYQLIW |
| 88 | TYFCAANDGGLS | 88 | TYFCAASPDSNYQLIW |
| 89 | TYFCAASAGSNYQLIW | 89 | TYFCAASAGSNYQLIW |
| 90 | TYFCAASEGAGGLS | 90 | TYFCAASPGGLS |
| 91 | TYFCAASRWTGGLS | 91 | TYFCAASNTGGLS |
| 92 | TYFCAQMDSNYQLIW | 92 | TYFCAATTNTGGLS |
| 93 | TYFCAATKGSNYQLIW | 93 | TYFCAAYGHSNYQLIW |
| 94 | TYFCAATSNTGGLS | 94 | TYFCAASGGGNYQLIW |
| 95 | TYFCAASGAYSNYQLIW | 95 | TYFCAAKGDSNYQLIW |
| 96 | TYFCAVSNTGGLS | 96 | TYFCAASERGNYQLIW |
| 97 | TYFCAASAKRVSNYQLIW | 97 | TYFCAASTGGLS |
| 98 | TYFCAANNGYQLIW | 98 | TYFCAASAEIYQLIW |
| 99 | TYFCAARNTGGLS | 99 | TYFCAASATSNYQLIW |
| 100 | TYFCAASARNTGGLS | 100 | TYFCAAEDSNYQLIW |
| 101 | TYFCAAGDSNYQLIW | 101 | TYFCAASEGNYQLIW |
| 102 | TYFCAASGGSNYQLIW |  |  |
| 103 | TYFCAADSNYQLIW |  |  |
| 104 | TYFCAAAFSQLIW |  |  |
| 105 | TYFCAAYSHSNYQLIW |  |  |

**Supplementary Data 2. Amino acid sequences of TCRα CDR3 regions shown in Supplementary Figure 3a.**

| 1 |  | TYFCAARGGSNYQLIW |
| --- | --- | --- |
| 2 |  | TYFCAAHPRNSNYQLIW |
| 3 |  | TYFCAAPGTGGLS |
| 4 |  | TYFCAAFPDSNYQLIW |
| 5 |  | TYFCAAKPVGNYQLIW |
| 6 |  | TYFCAASAGNYQLIW |
| 7 |  | TYFCAASAGSNYQLIW |
| 8 |  | TYFCAASANSNYQLIW |
| 9 |  | TYFCAASGRVDSNYQLIW |
| 10 |  | TYFCAVGSDYQLIW |
| 11 |  | TYFCAVFRGDSNYQLIW |
| 12 |  | TYFCAASFDQLIW |
| 13 |  | TYFCADRRTGGLS |
| 14 |  | TYFCAASERLGQLIW |
| 15 |  | TYFCAASGAYSNYQLIW |
| 16 |  | TYFCAASDSNYQLIW |
| 17 |  | TYFCAAMDSNYQLIW |
| 18 |  | TYFCAADAWSNYQLIW |
| 19 |  | TYFCAADKYSNYQLIW |
| 20 |  | TYFCAASAMGTGGLS |
| 21 |  | TYFCAASAWYTGGLS |
| 22 |  | TYFCAASAEGSNLIW |
| 23 |  | TYFCAADTFGSNYQLIW |
| 24 |  | TYFCAASAVGYQLIW |
| 25 |  | TYFCAASSNHYQLIW |
| 26 |  | TYFCAASPRGGGLS |
| 27 |  | TYFCAAYSHSNYQLIW |
| 28 |  | TYFCAASGGGNYQLIW |
| 29 |  | TYFCAATDNTGGLS |
| 30 |  | TYFCAASSNTGGLS |
| 31 |  | TYFCAAIDKGRSNYQLIW |
| 32 |  | TYFCAAEKSNYQLIW |
| 33 |  | TYFCAASAKRVSNYQLIW |
| 34 |  | TYFCAAELGNYQLIW |
| 35 |  | TYFCAANWKGNYQLIW |
| 36 |  | TYFCAATGGNYQLIW |
| 37 |  | TYFCAASRGGLS |
| 38 |  | TYFCAASSSNYQLIW |
| 39 |  | TYFCGEDSNYQLIW |
| 40 |  | TYFCAASETGGLS |
| 41 |  | TYFCAASGDAGGLS |
| 42 |  | TYFCAARDSNYQLIW |
| 43 |  | TYFCAASPMNTGGLS |
| 44 |  | TYFCAATGTGGLS |
| 45 |  | TYFCAASPPWHYQLIW |
| 46 |  | TYFCAAAFLNSNYQLIW |
| 47 |  | TYFCAQMDSNYQLIW |
| 48 |  | TYFCAATSNTGGLS |
| 49 |  | TYFCAASEGAGGLS |
| 50 |  | TYFCAASARSNYQLIW |

**Supplementary Data 3. Amino acid sequences of TCRα CDR3 regions shown in Supplementary Figure 3b and c.**

| **3b** Foxp3^GFP-^ | | **3c** Foxp3^GFP+^ | |
| --- | --- | --- | --- |
| 1 | TYFCAASEGAGGLS | 1 | TYFCAASGGNYQLIW |
| 2 | TYFCAARDSNYQLIW | 2 | TYFCAAMNSNYQLIW |
| 3 | TYFCAASARSNYQLIW | 3 | TYFCAANDSNYQLIW |
| 4 | TYFCAATSNTGGLS | 4 | TYFCAASAKRVSNYQLIW |
| 5 | TYFCAASDSNYQLIW | 5 | TYFCAASPMNTGGLS |
| 6 | TYFCAATGTGGLS | 6 | TYFCAASEGAGGLS |
| 7 | TYFCAQMDSNYQLIW | 7 | TYFCAASARSNYQLIW |
| 8 | TYFCAATDNTGGLS | 8 | TYFCAQMDSNYQLIW |
| 9 | TYFCAATGGNYQLIW | 9 | TYFCAATPSNYQLIW |
| 10 | TYFCAAAFLNSNYQLIW | 10 | TYFCAASPENTGGLS |
| 11 | TYFCAASFVDSNYQLIW | 11 | TYFCAASEPNYQLIW |
| 12 | TYFCAADGDSNYQLIW | 12 | TYFCAAMDSNYQLIW |
| 13 | TYFCAAKSNYQLIW | 13 | TYFCAASAVSNYQLIW |
| 14 | TYFCAANTGGLS | 14 | TYFCAASGQSNYQLIW |
| 15 | TYFCAAFTDSNYQLIW | 15 | TYFCAASGWAGGLS |
| 16 | TYFCAASANSNYQLIW | 16 | TYFCAATSSNYQLIW |
| 17 | TYFCAASRWTGGLS | 17 | TYFCAASAGGSNYQLIW |
| 18 | TYFCAASVGRLS | 18 | TYFCAASGGGNYQLIW |
| 19 | TYFCAASPPWHYQLIW | 19 | TYFCAAAPDSNYQLIW |
| 20 | TYFCAASGDAGGLS | 20 | TYFCAARGWSNYQLIW |
| 21 | TYFCAAYSHSNYQLIW | 21 | TYFCAASERGNYQLIW |
| 22 | TYFCAASPMNTGGLS | 22 | TYFCAAETGGLS |
| 23 | TYFCAASRGGLS | 23 | TYFCAANDGGLS |
| 24 | TYFCGEDSNYQLIW | 24 | TYFCAASDSNYQLIW |
| 25 | TYFCADMDSNYQLIW | 25 | TYFCAASGDQLIW |
| 26 | TYFCAASGAGSNYQLIW | 26 | TYFCAASPSNYQLIW |
| 27 | TYFCAASGRVDSNYQLIW | 27 | TYFCAAAPNYQLIW |
| 28 | TYFCAASSSNYQLIW | 28 | TYFCAAEKSNYQLIW |
| 29 | TYFCAASAVWTGGLS | 29 | TYFCAAIGGVDSNYQLIW |
| 30 | TYFCAASETGGLS | 30 | TYFCAAREGSNYQLIW |
| 31 | TYFCAASPRGGGLS | 31 | TYFCAASANSNYQLIW |
| 32 | TYFCAAFPDSNYQLIW | 32 | TYFCAASGDAGGLS |
| 33 | TYFCAANWKGNYQLIW | 33 | TYFCAASGTGGLS |
| 34 | TYFCAASESNYQLIW | 34 | TYFCAASPDNTGGLS |
| 35 | TYFCAHFMDSNYQLIW | 35 | TYFCAASPSWDSNYQLIW |
| 36 | TYFCAASFGLS | 36 | TYFCAATGGNYQLIW |
| 37 | TYFCAASAGSNYQLIW | 37 | TYFCAATRGSNYQLIW |
| 38 | TYFCAASGDQLIW | 38 | TYFCASMDSNYQLIW |
| 39 | TYFCAAALNSNYQLIW | 39 | TYFCAAEDSTGGLS |
| 40 | TYFCAASAVGYQLIW | 40 | TYFCAAKLGGNYQLIW |
| 41 | TYFCAASAVSNYQLIW | 41 | TYFCAASAEIYQLIW |
| 42 | TYFCAASGILSNYQLIW | 42 | TYFCAASAFDYQLIW |
| 43 | TYFCAASAWYTGGLS | 43 | TYFCAASAISNYQLIW |
| 44 | TYFCAAEKSNYQLIW | 44 | TYFCAASESNYQLIW |
| 45 | TYFCAASLGGLS | 45 | TYFCAASGMDSNYQLIW |
| 46 | TYFCAASGLDSNYQLIW | 46 | TYFCAASRGGLS |
| 47 | TYFCAASAKRVSNYQLIW | 47 | TYFCAASRPSNYQLIW |
| 48 | TYFCAASALATGGLS | 48 | TYFCAAEDSNYQLIW |
| 49 | TYFCAAELGNYQLIW | 49 | TYFCAAFTDSNYQLIW |
| 50 | TYFCAAFKRSNYQLIW | 50 | TYFCAAGGLS |
| 51 | TYFCAADAWSNYQLIW | 51 | TYFCAAGTGGLS |
| 52 | TYFCAAIDKGRSNYQLIW | 52 | TYFCAAKDSNYQLIW |
| 53 | TYFCAAMDSNYQLIW | 53 | TYFCAAMSSNYQLIW |
| 54 | TYFCAASPHSNYQLIW | 54 | TYFCAARDSNYQLIW |
| 55 | TYFCAASGGGNYQLIW | 55 | TYFCAARLNSNYQLIW |
| 56 | TYFCAASSNHYQLIW | 56 | TYFCAARPNSNYQLIW |
| 57 | TYFCAASGQRLS | 57 | TYFCAASAGSNYQLIW |
| 58 | TYFCAASGTHSNYQLIW | 58 | TYFCAASGAYSNYQLIW |
| 59 | TYFCAASSNTGGLS | 59 | TYFCAASGWDSNYQLIW |
| 60 | TYFCAASAMGTGGLS | 60 | TYFCAASSPHSNYQLIW |
| 61 | TYFCAADKYSNYQLIW | 61 | TYFCAATSNTGGLS |
| 62 | TYFCAATGSGDSNYQLIW | 62 | TYFCAAYRKNYQLIW |
| 63 | TYFCAASEGLS | 63 | TYFCAQLNTGGLS |
| 64 | TYFCAASAEGSNLIW | 64 | TYFCAVNFVDSNYQLIW |
| 65 | TYFCAARFNSNYQLIW | 65 | TYFCAADSNYQLIW |
| 66 | TYFCAASAFDYQLIW | 66 | TYFCAAELGNYQLIW |
| 67 | TYFCAADTFGSNYQLIW | 67 | TYFCAAEVWGSNYQLIW |
| 68 | TYFCAASGNTGGLS | 68 | TYFCAAGDTGGLS |
| 69 | TYFCAASAGNYQLIW | 69 | TYFCAAGPNSNYQLIW |
| 70 | TYFCAARGGSNYQLIW | 70 | TYFCAAHPRNSNYQLIW |
| 71 | TYFCAASAKGSNYQLIW | 71 | TYFCAAKGDSNYQLIW |
| 72 | TYFCAGNTGGLS | 72 | TYFCAAQLGGNYQLIW |
| 73 | TYFCAASAGDSNYQLIW | 73 | TYFCAARERDSNYQLIW |
| 74 | TYFCAAHPRNSNYQLIW | 74 | TYFCAARFGGNYQLIW |
| 75 | TYFCAASVDSNYQLIW | 75 | TYFCAARGGSNYQLIW |
| 76 | TYFCAASANTGGLS | 76 | TYFCAARPGGLS |
| 77 | TYFCAAGDSNYQLIW | 77 | TYFCAASADNYQLIW |
| 78 | TYFCAANSNYQLIW | 78 | TYFCAASAQEGLS |
| 79 | TYFCAATRLS | 79 | TYFCAASATGGLS |
| 80 | TYFCAAPGTGGLS | 80 | TYFCAASDGGLS |
| 81 | TYFCAAGPLYQLIW | 81 | TYFCAASDNSNYQLIW |
| 82 | TYFCAARDTGGLS | 82 | TYFCAASEDQLIW |
| 83 | TYFCAASTGSNYQLIW | 83 | TYFCAASFDQLIW |
| 84 | TYFCAAKAASNYQLIW | 84 | TYFCAASFNSNYQLIW |
| 85 | TYFCAVFRGDSNYQLIW | 85 | TYFCAASFSNYQLIW |
| 86 | TYFCAASGAYSNYQLIW | 86 | TYFCAASFVDSNYQLIW |
| 87 | TYFCAASSPHSNYQLIW | 87 | TYFCAASGDNYQLIW |
| 88 | TYFCAARRNSNYQLIW | 88 | TYFCAASGESNYQLIW |
| 89 | TYFCAASAYDYQLIW | 89 | TYFCAASGNSNYQLIW |
| 90 | TYFCAASDNSNYQLIW | 90 | TYFCAASGSDSNYQLIW |
| 91 | TYFCAAMNSNYQLIW | 91 | TYFCAASLNSNYQLIW |
| 92 | TYFCAANGGGLS | 92 | TYFCAASPYSNYQLIW |
| 93 | TYFCALAGGQLIW | 93 | TYFCAASRAGSNYQLIW |
| 94 | TYFCAVGSDYQLIW | 94 | TYFCAASTNSNYQLIW |
| 95 | TYFCADRRTGGLS | 95 | TYFCAASVPHSNYQLIW |
| 96 | TYFCALDSNYQLIW | 96 | TYFCAATPENTGGLS |
| 97 | TYFCAVGENSNYQLIW | 97 | TYFCAAYIRNSNYQLIW |
| 98 | TYFCRPGDSNYQLIW | 98 | TYFCAEESNYQLIW |
| 99 | TYFCAASPSNYQLIW | 99 | TYFCAHFMDSNYQLIW |
| 100 | TYFCAASERLGQLIW | 100 | TYFCALGRNSNYQLIW |
| 101 | TYFCAASFDQLIW | 101 | TYFCANYQLIW |
| 102 | TYFCAASLSNYQLIW | 102 | TYFCASDSNYQLIW |
| 103 | TYFCAASGEATGGLS | 103 | TYFCAWDSNYQLIW |
| 104 | TYFCAASLFSGTGGLS | 104 | TYFCGHTGGLS |
| 105 | TYFCAASNDYQLIW | 105 | TYFCRDSNYQLIW |
| 106 | TYFCAARESFGNYQLIW | 106 | TYFCWVDSNYQLIW |
| 107 | TYFCAASAQRGLS | 107 | TYFCAASALDSNYQLIW |
| 108 | TYFCAASAWKNYQLIW | 108 | TYFCAARESNYQLIW |
| 109 | TYFCAASATDYQLIW | 109 | TYFCAARAPFNYQLIW |
| 110 | TYFCAASDLGSNYQLIW | 110 | TYFCAASPDSNYQLIW |
| 111 | TYFCAASDGAGGLS | 111 | TYFCAASDSKGDSNYQLIW |
| 112 | TYFCAAKRSGNYQLIW | 112 | TYFCAASYSNYQLIW |
| 113 | TYFCAAKAGGNYQLIW | 113 | TYFCAATPGGLS |
| 114 | TYFCAAEGPHSNYQLIW | 114 | TYFCAAPVLTGGLS |
| 115 | TYFCAAKPVGNYQLIW | 115 | TYFCAASEGNYQLIW |
| 116 | TYFCAAGGGTGGLS | 116 | TYFCAASGGWDSNYQLIW |
| 117 | TYFCAANVGRSNYQLIW | 117 | TYFCAADTGGLS |
| 118 | TYFCAANVGGNYQLIW | 118 | TYFCAAKTESNYQLIW |
| 119 | TYFCAASDSKGDSNYQLIW | 119 | TYFCAARNSNYQLIW |
| 120 | TYFCAASWGRGGLS | 120 | TYFCAASAAGGSNYQLIW |
| 121 | TYFCAASRAGSNYQLIW | 121 | TYFCAASAESNYQLIW |
| 122 | TYFCAAYIRNSNYQLIW | 122 | TYFCAASAFSNYQLIW |
| 123 | TYFCAATPGGLS | 123 | TYFCAASASNYQLIW |
| 124 | TYFCAASWNDYQLIW | 124 | TYFCAASDVRGDSNYQLIW |
| 125 | TYFCAAIDDYQLIW | 125 | TYFCAASFGLS |
| 126 | TYFCAASAGGSNYQLIW | 126 | TYFCAASMDSNYQLIW |
| 127 | TYFWTYVSNYQLIW | 127 | TYFCAASRGDNYQLIW |
| 128 | TYFCAAKDSNYQLIW | 128 | TYFCAASWISNYQLIW |
| 129 | TYFCAASGGSNYQLIW | 129 | TYFCAASYPGSNYQLIW |
| 130 | TYFCAAIRGSSNYQLIW | 130 | TYFCAASGNTGGLS |
| 131 | TYFCAADDKNYQLIW | 131 | TYFCAASRAHQLIW |
| 132 | TYFCAASEDQLIW | 132 | TYFCAAHLRNSNYQLIW |
| 133 | TYFCAWDSNYQLIW | 133 | TYFCAATNTGGLS |
| 134 | TYFCAAVPVNYQLIW | 134 | TYFCAEGSNYQLIW |
| 135 | TYFCAASEGNYQLIW | 135 | TYFCAASAQGNYQLIW |
| 136 | TYFCAASEGSNYQLIW | 136 | TYFCGEDSNYQLIW |
| 137 | TYFCAAMGGVDSNYQLIW | 137 | TYFCAADENNYQLIW |
| 138 | TYFCAASGNSNYQLIW | 138 | TYFCAAHFRNSNYQLIW |
| 139 | TYFCAASNKGNYQLIW | 139 | TYFCAAMGGVDSNYQLIW |
| 140 | TYFCAASGECSNYQLIW | 140 | TYFCAASALFHYQLIW |
| 141 | TYFCAAKGGTGGLS | 141 | TYFCAASRGGGLS |
| 142 | TYFCAAGSYSNYQLIW | 142 | TYFCAAKEGLS |
| 143 | TYFCAASGTGGLS | 143 | TYFCAASDVKGDSNYQLIW |
| 144 | TYFCAARGTGYSNYQLIW | 144 | TYFCAASLDSNYQLIW |
| 145 | TYFCAAAKGGLS | 145 | TYFCAASRNNGGLS |
| 146 | TYFCAARESNYQLIW | 146 | TYFCAAFQGNSNYQLIW |
| 147 | TYFCAASEDSNYQLIW | 147 | TYFCAASAGDSNYQLIW |
| 148 | TYFCAASAHSNYQLIW | 148 | TYFCANEDSNYQLIW |
| 149 | TYFCAATEGNYQLIW | 149 | TYFCAALKDSNYQLIW |
| 150 | TYFCAASGVGSNYQLIW | 150 | TYFCAARVGGLS |
| 151 | TYFCAARDYSNYQLIW |  |  |
| 152 | TYFCAASVNYQLIW |  |  |
| 153 | TYFCAGSNYQLIW |  |  |
| 154 | TYFCAATEGLS |  |  |
| 155 | TYFCAATPGELS |  |  |
| 156 | TYFCAASRDSNYQLIW |  |  |
| 157 | TYFCAASAPPFYSNYQLIW |  |  |
| 158 | TYFCAANPHTGGLS |  |  |
| 159 | TYFCAASNTGGLS |  |  |
| 160 | TYFCAASAQGNYQLIW |  |  |
| 161 | TYFCAAHNSNYQLIW |  |  |
| 162 | TYFCAASNSNYQLIW |  |  |
| 163 | TYFCAASGWAGGLS |  |  |
| 164 | TYFCAASPENTGGLS |  |  |
| 165 | TYFCAADESNYQLIW |  |  |
| 166 | TYFCAASAKSGNYQLIW |  |  |
| 167 | TYFCAAKGSNYQLIW |  |  |
| 168 | TYFCAAEGGLLDSNYQLIW |  |  |
| 169 | TYFCAACPGGLS |  |  |
| 170 | TYFCAAMEDYQLIW |  |  |
| 171 | TYFCAASRAHQLIW |  |  |
| 172 | TYFCAARPMNTGGLS |  |  |
| 173 | TYFCAARNSNYQLIW |  |  |
| 174 | TYFCAARRWSNYQLIW |  |  |
| 175 | TYFCAARVGGLS |  |  |
| 176 | TYFCAAIKHSNYQLIW |  |  |
| 177 | TYFCAASGDSNYQLIW |  |  |
| 178 | TYFCAARMDSNYQLIW |  |  |
| 179 | TYFCAASLDSNYQLIW |  |  |
| 180 | TYFCAAGKMFQLIW |  |  |
| 181 | TYFCAASAYGTGGLS |  |  |
| 182 | TYFCAARPLNTGGLS |  |  |
| 183 | TYFCAASFSDSNYQLIW |  |  |
| 184 | TYFCAANDWDSNYQLIW |  |  |
| 185 | TYFCAASLNTGGLS |  |  |
| 186 | TYFCAARSGGLS |  |  |
| 187 | TYFCAADTRNYQLIW |  |  |
| 188 | TYFCAATNTGGLS |  |  |
| 189 | TYFCAARRSNYQLIW |  |  |
| 190 | TYFCAAFQGNSNYQLIW |  |  |
| 191 | TYFCAASASNYQLIW |  |  |
| 192 | TYFCAASPDSNYQLIW |  |  |
| 193 | TYFCAASDVKGDSNYQLIW |  |  |
| 194 | TYFCAASATGGLS |  |  |
| 195 | TYFCAASVGGWDYQLIW |  |  |
| 196 | TYFCAASTDSNYQLIW |  |  |
| 197 | TYFCAAGVLYQLIW |  |  |
| 198 | TYFCAAMTDYQLIW |  |  |
| 199 | TYFCAAFLSNYQLIW |  |  |
| 200 | TYFCAASWGNYQLIW |  |  |
| 201 | TYFCAGAGGLS |  |  |
| 202 | TYFCAASGASNYQLIW |  |  |
| 203 | TYFCAASGSADSNYQLIW |  |  |
| 204 | TYFCAALWDTGGLS |  |  |
| 205 | TYFCAAEDSTGGLS |  |  |
| 206 | TYFCAAHDSNYQLIW |  |  |
| 207 | TYFCAASAKSNYQLIW |  |  |
| 208 | TYFCAASAKLGQLIW |  |  |

**Supplementary Data 4. Amino acid sequences of TCRα CDR3 regions shown in Figure 3a, and Supplementary Figure 5a.**

| 1 | TYFCAARDSNYQLIW |
| --- | --- |
| 2 | TYFCAASESNYQLIW |
| 3 | TYFCAASASNYQLIW |
| 4 | TYFCAASSSNYQLIW |
| 5 | TYFCAARESNYQLIW |
| 6 | TYFCAASANSNYQLIW |
| 7 | TYFCAASDSNYQLIW |
| 8 | TYFCAAMDSNYQLIW |
| 9 | TYFCAASADYQLIW |
| 10 | TYFCAARGWTGGLS |
| 11 | TYFCAASGSNYQLIW |
| 12 | TYFCAASAGSNYQLIW |
| 13 | TYFCAASAVWTGGLS |
| 14 | TYFCAAMINSNYQLIW |
| 15 | TYFCAASAISNYQLIW |
| 16 | TYFCAAHIRNYQLIW |
| 17 | TYFCAAANTGGLS |
| 18 | TYFCAAYTGGLS |
| 19 | TYFCAARYLNYQLIW |
| 20 | TYFCAASRDTGGLS |

**Supplementary Data 5. Amino acid sequences of TCRα CDR3 regions from autoreactive hybridomas.**

| 1 | TYFCAAFPDSNYQLIW |
| --- | --- |
| 2 | TYFCAAVGDQLIW |
| 3 | TYFCAASVGGWDYQLIW |
| 4 | TYFCAATSPSNYQLIW |
| 5 | TYFCAASEGWDYQLIW |
| 6 | TYFCAASALFHYQLIW |
| 7 | TYFCAASPMNT |
| 8 | TYFCAGEGNSNYQLIW |
| 9 | TYFCAAGDSNYQLIW |
| 10 | TYFCAARRWDSNYQLIW |
| 11 | TYFCAANTG |
| 12 | TYFCAARSAGG |
| 13 | TYFCAAREMDSNYQLIW |
| 14 | TYFCAAFNYQLIW |
| 15 | TYFCAAMRNYQLIW |
| 16 | TYFCAAMEGSNYQLIW |
| 17 | TYFCAASLADSNYQLIW |
| 18 | TYFCAATGNYQLIW |
| 19 | TYFCAARRWSNYQLIW |
| 20 | TYFCAASAGDLLIW |
| 21 | TYFCAASEKGGNYQLIW |
| 22 | TYFCAADSNYQLIW |
| 23 | TYFCAASTPSNYQLIW |
| 24 | TYFCAASEDQLIW |
| 25 | TYFCAAGGGTGG |
| 26 | TYFCAARIRDSNYQLIW |
| 27 | TYFCAASATGGLS |
| 28 | TYFCAAYDGLDSNYQLIW |
| 29 | TYFCAAPHMDSNYQLIW |
| 30 | TYFCAAAWRNSNYQLIW |
| 31 | TYFCAQMDSNYQLIW |
| 32 | TYFCAAKLGGNYQLIW |
| 33 | TYFCAASGAYSNYQLIW |
| 34 | TYFCAASGRRISPSNYQLIW |
| 35 | TYFCAASSSYQLIW |
| 36 | TYFCAASTASNYQLIW |
| 37 | TYFCAASAWFSNYQLIW |
| 38 | TYFCAASADYQLIW |
| 39 | TYFCAARGWTGGLS |
| 40 | TYFCAASGSNYQLIW |
| 41 | TYFCAASAGSNYQLIW |
| 42 | TYFCAASAVWTGGLS |
| 43 | TYFCAAMINSNYQLIW |
| 44 | TYFCAASMKSNYQLIW |
| 45 | TYFCAAMRGGLS |
| 46 | TYFCAASAISNYQLIW |
| 47 | TYFCAASGWNSNYQLIW |
| 48 | TYFCAAIWGSNYQLIW |
| 49 | TYFCAAHIRNYQLIW |
| 50 | TYFCAAKGDSNYQLIW |
| 51 | TYFCAASERSNYQLIW |
| 52 | TYFCAARSDSNYQLIW |
| 53 | TYFCAASASNYQLIW |
| 54 | TYFCTILSNYQLIW |
| 55 | TYFCAASALNNYQLIW |
| 56 | TYFCAASEGSNYQLIW |
| 57 | TYFCAAPLRYSNYQLIW |
| 58 | TYFCAAALRNSNYQLIW |
| 59 | TYFCAGKEARNYQLIW |
| 60 | TYFCAARPDSNYQLIW |
| 61 | TYFCAAPINHYQLIW |
| 62 | TYFCAASGTGGLS |
| 63 | TYFCAASELRNYQLIW |
| 64 | TYFCAAPARDSNYQLIW |
| 65 | TYFCAATGGHYQLIW |
| 66 | TYFCAATSYTGGLS |
| 67 | TYFCAAFTDSNYQLIW |
| 68 | TYFCAAANTGGLS |
| 69 | TYFCAASATGLS |
| 70 | TYFCAASSSNYQLIW |
| 71 | TYFCAARYLNYQLIW |
| 72 | TYFCAASGRADSNYQLIW |
| 73 | TYFCAASRDTGGLS |
| 74 | TYFCAAYTGGLS |
| 75 | TYFCAARWTGGLS |
| 76 | TYFCARKWSGSNYQLIW |
| 77 | TYFCAASNTGGLS |
| 78 | TYFCAAEDSTGGLS |
| 79 | TYFCAAPRIYSNYQLIW |
| 80 | TYFCAARSS |
| 81 | TYFCAPNTGGLS |
| 82 | TYFCAASGVGSNYQLIW |
| 83 | TYFCAASWKGSNYQLIW |
| 84 | TYFCAASEVRNYQLIW |
| 85 | TYFCAADGDSNYQLIW |
| 86 | TYFCAARNSNYQLIW |
| 87 | TYFCAAVGSNYQLIW |
| 88 | TYFCAAWDSNYQLIW |
| 89 | TYFCAASFVDSNYQLLW |
| 90 | TYFCAAALNSNYQLIW |
| 91 | TYFCAASAQLGNYQLIW |
| 92 | TYFCAASRWTGGLS |
| 93 | TYFCAASPSWDSNYQLIW |
| 94 | TYFCAARPMNTGGLS |
| 95 | TYFCAARDTGGLS |
| 96 | TYFCAASRGGGLS |
| 97 | TYFCAAFRGDSNYQLIW |
| 98 | TYFCAANDWDSNYQLIW |
| 99 | TYFCAAPPSYGDSNYQLIW |
| 100 | TYFCAQLYSNYQLIW |
| 101 | TYFCAARMDSNYQLIW |
| 102 | TYFCAASNYQLIW |
| 103 | TYFCAASFSDSNYQLIW |
| 104 | TYFCAAPGTGGLS |
| 105 | TYFCAAGPKSNYQLIW |
| 106 | TYFCALMRGDSNYQLIW |
| 107 | TYFCAASARNYQLIW |
| 108 | TYFCAASDGSSNYQLIW |
| 109 | TYFCAARPTVILEGLS |
| 110 | TYFCAASGGGNYQLIW |
| 111 | TYFCAASMRNSNYQLIW |
| 112 | TYFCAASVGDSNYQLIW |
| 113 | TYFCAARGVGSNYQLIW |
| 114 | TYFCAARGGGLS |
| 115 | TYFCAGTKGSNYQLIW |
| 116 | TYFCAGGANSNYQLIW |
| 117 | TYFCAGAGGLS |
| 118 | TYFCAATSNTGGLS |
| 119 | TYFCAATNTGGLS |
| 120 | TYFCAATGTGGLS |
| 121 | TYFCAATGGNYQLIW |
| 122 | TYFCAASVWWGNYQLIW |
| 123 | TYFCAASSQRLS |
| 124 | TYFCAASRGHYQLIW |
| 125 | TYFCAASRGGLS |
| 126 | TYFCAASRDLS |
| 127 | TYFCAASPMNTGGLS |
| 128 | TYFCAASPHSNYQLIW |
| 129 | TYFCAASPENTGGLS |
| 130 | TYFCAASNVGNYQLIW |
| 131 | TYFCAASNSNYQLIW |
| 132 | TYFCAASLNTGGLS |
| 133 | TYFCAASGNTGGLS |
| 134 | TYFCAASFVDSNYQLIW |
| 135 | TYFCAASFAGTGGLS |
| 136 | TYFCAASESNYQLIW |
| 137 | TYFCAASDTGGLS |
| 138 | TYFCAASDSNYQLIW |
| 139 | TYFCAASDGSNYQLIW |
| 140 | TYFCAASAVSNYQLIW |
| 141 | TYFCAASASSNYQLIW |
| 142 | TYFCAASAQSNYQLIW |
| 143 | TYFCAASANSNYQLIW |
| 144 | TYFCAASAHSNYQLIW |
| 145 | TYFCAARVNTGGLS |
| 146 | TYFCAARHYQLIW |
| 147 | TYFCAARESNYQLIW |
| 148 | TYFCAARDSNYQLIW |
| 149 | TYFCAANTGGLS |
| 150 | TYFCAANSNYQLIW |
| 151 | TYFCAANDSNYQLIW |
| 152 | TYFCAAMDSNYQLIW |
| 153 | TYFCAAKYGGNYQLIW |
| 154 | TYFCAAKPVGNYQLIW |
| 155 | TYFCAAKGSNYQLIW |
| 156 | TYFCAAKAGGNYQLIW |
| 157 | TYFCAAKAASNYQLIW |
| 158 | TYFCAAIDKARSNYQLIW |
| 159 | TYFCAAADGNYQLIW |

**Supplementary Data 6. Amino acid sequences of TCRα chains CDR3 regions shown in Figure 3d.**

| 1 |  | TYFCAAYSHSNYQLIW |
| --- | --- | --- |
| 2 |  | TYFCAAAFSQLIW |
| 3 |  | TYFCAARMDSNYQLIW |
| 4 |  | TYFCAADSNYQLIW |
| 5 |  | TYFCAASPENTGGLS |
| 6 |  | TYFCAASGGSNYQLIW |
| 7 |  | TYFCAASARSNYQLIW |
| 8 |  | TYFCAAGDSNYQLIW |
| 9 |  | TYFCAASARNTGGLS |
| 10 |  | TYFCAARAHSNYQLIW |
| 11 |  | TYFCAATGGNYQLIW |
| 12 |  | TYFCAARESNYQLIW |
| 13 |  | TYFCAARNTGGLS |
| 14 |  | TYFCAANNGYQLIW |
| 15 |  | TYFCAASAKRVSNYQLIW |
| 16 |  | TYFCAASLNTGGLS |
| 17 |  | TYFCAAWSNYQLIW |
| 18 |  | TYFCAVSNTGGLS |
| 19 |  | TYFCAASAVGYQLIW |
| 20 |  | TYFCAASEGNYQLIW |
| 21 |  | TYFCAASGAYSNYQLIW |
| 22 |  | TYFCAASANSNYQLIW |
| 23 |  | TYFCAATSNTGGLS |
| 24 |  | TYFCAATKGSNYQLIW |
| 25 |  | TYFCAASGNTGGLS |
| 26 |  | TYFCAASRGGLS |
| 27 |  | TYFCAASHGTGGLS |
| 28 |  | TYFCAASGTGGLS |
| 29 |  | TYFCAQMDSNYQLIW |
| 30 |  | TYFCAAWTHSNYQLIW |
| 31 |  | TYFCAASGDQLIW |
| 32 |  | TYFCAASRWTGGLS |
| 33 |  | TYFCAASGANTGGLS |
| 34 |  | TYFCAASEGAGGLS |
| 35 |  | TYFCAASGGLS |
| 36 |  | TYFCAASNTGGLS |
| 37 |  | TYFCAASESNYQLIW |
| 38 |  | TYFCAAMDSNYQLIW |
| 39 |  | TYFCAANTGGLS |
| 40 |  | TYFCAAWRHSNYQLIW |
| 41 |  | TYFCAASPDSNYQLIW |
| 42 |  | TYFCAASFVDSNYQLIW |
| 43 |  | TYFCAASDSNYQLIW |
| 44 |  | TYFCAAKDSNYQLIW |
| 45 |  | TYFCAASPMNTGGLS |
| 46 |  | TYFCAASAHSNYQLIW |
| 47 |  | TYFCAAKGSNYQLIW |
| 48 |  | TYFCAANSNYQLIW |
| 49 |  | TYFCAASAVWTGGLS |
| 50 |  | TYFCAARDSNYQLIW |

**Supplementary Data 7. Amino acid sequences of TCRα chains CDR3 regions shown in Supplementary Figure 12a.**

| 1 | TYFCAADSNYQLIW |
| --- | --- |
| 2 | TYFCAGGNYQLIW |
| 3 | TYFCAASSYQLIW |
| 4 | TYFCAASVYQLIW |
| 5 | TYFCAATNYQLIW |
| 6 | TYFCGGSNYQLIW |
| 7 | TYFCAASGSNYQLIW |
| 8 | TYFCAVRNYQLIW |
| 9 | TYFCAAMNYQLIW |
| 10 | TYFCAASPYQLIW |
| 11 | TYFCAASTYQLIW |
| 12 | TYFCAASRSNYQLIW |
| 13 | TYFCAASFYYQLIW |
| 14 | TYFCAARDYQLIW |
| 15 | TYFCAARSYQLIW |
| 16 | TYFCAASRYQLIW |
| 17 | TYFCAPCNYQLIW |
| 18 | TYFCAADHYQLIW |
| 19 | TYFCVPSNYQLIW |
| 20 | TYFCAALNYQLIW |
| 21 | TYFCAASEMSNYQLIW |
| 22 | TYFCAARRYQLIW |
| 23 | TYFCAAKHYQLIW |
| 24 | TYFCAVSNYQLIW |
| 25 | TYFCAGDNYQLIW |
| 26 | TYFCAPTNYQLIW |
| 27 | TYFCAARAHSNYQLIW |
| 28 | TYFCAPRNYQLIW |
| 29 | TYFCAASDAGSNYQLIW |
| 30 | TYFCAAKYQLIW |
| 31 | TYFCAARYYQLIW |
| 32 | TYFCAASHYQLIW |
| 33 | TYFCAASDYQLIW |
| 34 | TYFCAARDHSNYQLIW |
| 35 | TYFCAAKNYQLIW |
| 36 | TYFCAASDMSNYQLIW |
| 37 | TYFCAANNYQLIW |
| 38 | TYFCAPGNYQLIW |
| 39 | TYFCAARLYQLIW |
| 40 | TYFCAASLYQLIW |
| 41 | TYFCAGSNYQLIW |
| 42 | TYFCAARSHSNYQLIW |
| 43 | TYFCAAGNYQLIW |
| 44 | TYFCAASAHSNYQLIW |
| 45 | TYFCAAFNYQLIW |
| 46 | TYFCAARHYQLIW |
| 47 | TYFCAADNYQLIW |
| 48 | TYFCAPSNYQLIW |
| 49 | TYFCAARNYQLIW |
| 50 | TYFCAASNYQLIW |

**Supplementary Data 8. Amino acid sequences of TCRα chains CDR3 regions shown in Supplementary Figure 12b.**

| 1 | TYFCAASNYQLIW |
| --- | --- |
| 2 | TYFCAARNYQLIW |
| 3 | TYFCAPSNYQLIW |
| 4 | TYFCAADNYQLIW |
| 5 | TYFCAARHYQLIW |
| 6 | TYFCAAFNYQLIW |
| 7 | TYFCAASAHSNYQLIW |
| 8 | TYFCAAGNYQLIW |
| 9 | TYFCAARSHSNYQLIW |
| 10 | TYFCAGSNYQLIW |
| 11 | TYFCAASLYQLIW |
| 12 | TYFCAARLYQLIW |
| 13 | TYFCAPGNYQLIW |
| 14 | TYFCAANNYQLIW |
| 15 | TYFCAASDMSNYQLIW |
| 16 | TYFCAAKNYQLIW |
| 17 | TYFCAARDHSNYQLIW |
| 18 | TYFCAASDYQLIW |
| 19 | TYFCAASHYQLIW |
| 20 | TYFCAARYYQLIW |
| 21 | TYFCAAKYQLIW |
| 22 | TYFCAASDAGSNYQLIW |
| 23 | TYFCAPRNYQLIW |
| 24 | TYFCAARAHSNYQLIW |
| 25 | TYFCAPTNYQLIW |
| 26 | TYFCAGDNYQLIW |
| 27 | TYFCAVSNYQLIW |
| 28 | TYFCAAKHYQLIW |
| 29 | TYFCAARRYQLIW |
| 30 | TYFCAASEMSNYQLIW |
| 31 | TYFCAALNYQLIW |
| 32 | TYFCVPSNYQLIW |
| 33 | TYFCAADHYQLIW |
| 34 | TYFCAPCNYQLIW |
| 35 | TYFCAASRYQLIW |
| 36 | TYFCAARSYQLIW |
| 37 | TYFCAARDYQLIW |
| 38 | TYFCAASRSNYQLIW |
| 39 | TYFCAASTYQLIW |
| 40 | TYFCAASPYQLIW |
| 41 | TYFCAAMNYQLIW |
| 42 | TYFCAVRNYQLIW |
| 43 | TYFCAASGSNYQLIW |
| 44 | TYFCGGSNYQLIW |
| 45 | TYFCAATNYQLIW |
| 46 | TYFCAASSYQLIW |
| 47 | TYFCAADSNYQLIW |
| 48 | TYFCAARFYQLIW |
| 49 | TYFCAASAYQLIW |
| 50 | TYFCAAMGSNYQLIW |
| 51 | TYFCAARRHSNYQLIW |
| 52 | TYFCAAENYQLIW |
| 53 | TYFCAAHNYQLIW |
| 54 | TYFCAAINYQLIW |
| 55 | TYFCAARTYQLIW |
| 56 | TYFCAANTGGLS |
| 57 | TYFCAARKYQLIW |
| 58 | TYFCAASIYQLIW |
| 59 | TYFCAANSNYQLIW |
| 60 | TYFCAASFYYQLIW |
| 61 | TYFCAASGNYQLIW |
| 62 | TYFCAASVYQLIW |
| 63 | TYFCAGGNYQLIW |
| 64 | TYFCASRNYQLIW |
| 65 | TYFCAAKSYQLIW |
| 66 | TYFCAARAYQLIW |
| 67 | TYFCAAKRYQLIW |
| 68 | TYFCPSNYQLIW |
| 69 | TYFCAARNSQLIW |

**Supplementary Data 9. Amino acid sequences of TCRα chains CDR3 regions shown in Figure 5a.**

| 1 | TYFCAADDYQLIW |
| --- | --- |
| 2 | TYFCAAENYQLIW |
| 3 | TYFCAAKNYQLIW |
| 4 | TYFCAAKRYQLIW |
| 5 | TYFCAALLHSNYQLIW |
| 6 | TYFCAAMNYQLIW |
| 7 | TYFCAARAHSNYQLIW |
| 8 | TYFCAARGHQLIW |
| 9 | TYFCAARHYQLIW |
| 10 | TYFCAARLYQLIW |
| 11 | TYFCAARNYQLIW |
| 12 | TYFCAARRHSNYQLIW |
| 13 | TYFCAASAHNNYQLIW |
| 14 | TYFCAASDYQLIW |
| 15 | TYFCAPSNYQLIW |

**Supplementary Data 10. Amino acid sequences of TCRα chains CDR3 regions shown in Figure 6c.**

| 1 | TYFCAASNYQLIW |
| --- | --- |
| 2 | TYFCAARNYQLIW |
| 3 | TYFCAPSNYQLIW |
| 4 | TYFCAADNYQLIW |
| 5 | TYFCAAFNYQLIW |
| 6 | TYFCAARHYQLIW |
| 7 | TYFCAASAHSNYQLIW |
| 8 | TYFCAAGNYQLIW |
| 9 | TYFCAARSHSNYQLIW |
| 10 | TYFCAGSNYQLIW |
| 11 | TYFCAASLYQLIW |
| 12 | TYFCAARLYQLIW |
| 13 | TYFCAPGNYQLIW |
| 14 | TYFCAANNYQLIW |
| 15 | TYFCAASDMSNYQLIW |
| 16 | TYFCAAKNYQLIW |
| 17 | TYFCAARDHSNYQLIW |
| 18 | TYFCAASDYQLIW |
| 19 | TYFCAASHYQLIW |
| 20 | TYFCAARYYQLIW |
| 21 | TYFCAASPDSNYQLIW |
| 22 | TYFCAASDAGSNYQLIW |
| 23 | TYFCAARAHSNYQLIW |
| 24 | TYFCAAKHYQLIW |
| 25 | TYFCAASEMSNYQLIW |
| 26 | TYFCAARDYQLIW |
| 27 | TYFCAASRSNYQLIW |
| 28 | TYFCAVRNYQLIW |
| 29 | TYFCAASGSNYQLIW |
| 30 | TYFCAATNYQLIW |
| 31 | TYFCAGGNYQLIW |
| 32 | TYFCAADSNYQLIW |
| 33 | TYFCAARDSNYQLIW |
| 34 | TYFCAASESNYQLIW |
| 35 | TYFCAANTGGLS |
| 36 | TYFCAAMDSNYQLIW |
| 37 | TYFCAASDMGNYQLIW |
| 38 | TYFCAASDSNYQLIW |
| 39 | TYFCAASAMSNYQLIW |
| 40 | TYFCAADGGSNYQLIW |
| 41 | TYFCAASASNYQLIW |
| 42 | TYFCAAGIYQLIW |
| 43 | TYFCAAKDSNYQLIW |
| 44 | TYFCAASARSNYQLIW |
| 45 | TYFCAASDTGGLS |
| 46 | TYFCAARGSNYQLIW |
| 47 | TYFCAASNTGGLS |
| 48 | TYFCAASNSNYQLIW |
| 49 | TYFCAARRSNYQLIW |
| 50 | TYFCAAGSNYQLIW |
| 51 | TYFCAASGTGGLS |
| 52 | TYFCAASDNYQLIW |
| 53 | TYFCAAHSNYQLIW |
| 54 | TYFCAASAFDYQLIW |
| 55 | TYFCAADESNYQLIW |
| 56 | TYFCAAKYQLIW |
| 57 | TYFCAPRNYQLIW |
| 58 | TYFCAPTNYQLIW |
| 59 | TYFCAGDNYQLIW |
| 60 | TYFCAVSNYQLIW |
| 61 | TYFCAARRYQLIW |
| 62 | TYFCAALNYQLIW |
| 63 | TYFCVPSNYQLIW |
| 64 | TYFCAADHYQLIW |
| 65 | TYFCAPCNYQLIW |
| 66 | TYFCAASRYQLIW |
| 67 | TYFCAARSYQLIW |
| 68 | TYFCAASFYYQLIW |
| 69 | TYFCAASTYQLIW |
| 70 | TYFCAASPYQLIW |
| 71 | TYFCAAMNYQLIW |
| 72 | TYFCGGSNYQLIW |
| 73 | TYFCAASVYQLIW |
| 74 | TYFCAASSYQLIW |
| 75 | TYFCAARSNYQLIW |
| 76 | TYFCAAIPDSNYQLIW |
| 77 | TYFCAANSNSNYQLIW |
| 78 | TYFCPGTGGLS |
| 79 | TYFCAAEGGGDSNYQLIW |
| 80 | TYFCAAKLYQLIW |
| 81 | TYFCAAKDYQLIW |
| 82 | TYFCAARWKGNYQLIW |
| 83 | TYFCAASLTQLIW |
| 84 | TYFCAASSNYQLIW |
| 85 | TYFCAASHQSNYQLIW |
| 86 | TYFCAAKAYQLIW |
| 87 | TYFCAARSKSNYQLIW |
| 88 | TYFCAARDTGGLS |
| 89 | TYFCAASYNQLIW |
| 90 | TYFCAASAYQLIW |
| 91 | TYFCAARWKSNYQLIW |
| 92 | TYFCAAHSNSNYQLIW |
| 93 | TYFCAASAGSNYQLIW |
| 94 | TYFCAAKYYQLIW |
| 95 | TYFCAAINYQLIW |
| 96 | TYFCAVDDSNYQLIW |
| 97 | TYFCAASQGSNYQLIW |
| 98 | TYFCAAEHYQLIW |
| 99 | TYFCAASRMNYQLIW |
| 100 | TYFCAANDNSNYQLIW |
| 101 | TYFCAAKKSGNYQLIW |
| 102 | TYFCAASVSNYQLIW |
| 103 | TYFCAASVRYQLIW |
| 104 | TYFCAADSGGDSNYQLIW |
| 105 | TYFCAAGGGRQLIW |
| 106 | TYFCAASAKSNYQLIW |
| 107 | TYFCAEDSNYQLIW |
| 108 | TYFCAASGYSNYQLIW |
| 109 | TYFCAASGGLS |
| 110 | TYFCAAERGNYQLIW |
| 111 | TYFCAAESNYQLIW |
| 112 | TYFCAAYSNYQLIW |
| 113 | TYFCAGDSNYQLIW |
| 114 | TYFCAANSNYQLIW |
| 115 | TYFCAASEGNYQLIW |
| 116 | TYFCAASKGSNYQLIW |
| 117 | TYFCAASEGSNYQLIW |


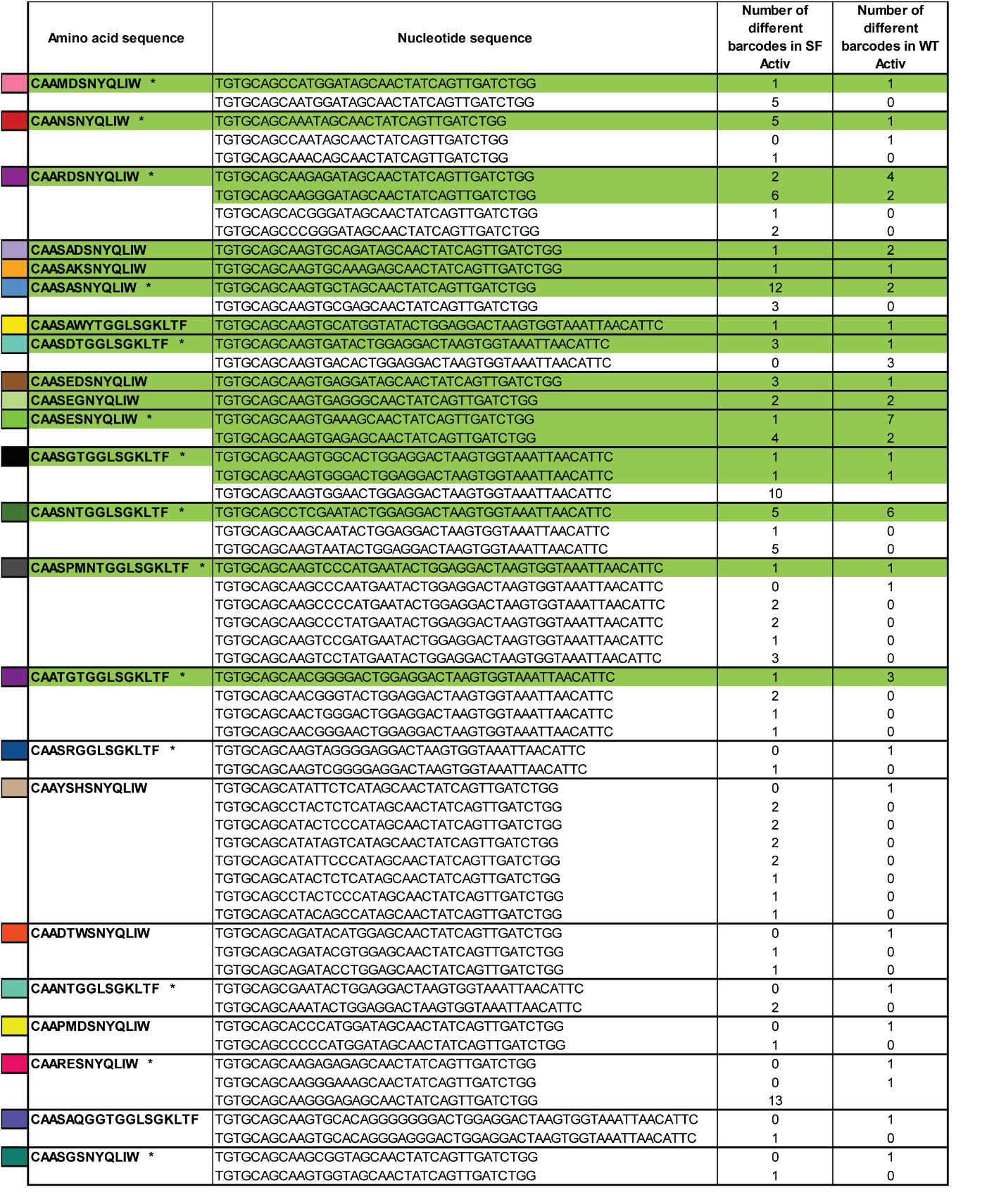
**Supplementary Data 11. Sequences of TCRα chains CDR3 regions (amino acid and nucleotide) shared by SfCD4**^+^**Foxp3^GFP-^ and CD4**^+^**Foxp3^GFP-^ cells** (Figure 7d-f). Frequencies of each shared TCR on studied subsets are also shown. Asterix marks TCRs also found on hybridomas established from SfCD4^+^Foxp3^GFP-^CD44^+^CD62L^-^ cells that responded *ex vivo* to autologous APCs. Green color marks TCRs encoded by identical nucleotide sequences.
