# Supplementary material for: Dormant pathogenic CD4+ T cells are prevalent in the peripheral repertoire of healthy mice
Source: Nat Commun. 2019 Oct 25;10:4882. doi: 10.1038/s41467-019-12820-3 (PMC6814812; doi:10.1038/s41467-019-12820-3)
Supplement: Supplementary file 4 — Reporting Summary [file 41467_2019_12820_MOESM4_ESM.pdf]

nature research

Corresponding author(s): Leszek Ignatowicz

Last updated by author(s): Sep 16, 2019

Reporting Summary

Nature Research wishes to improve the reproducibility of the work that we publish. This form provides structure for consistency and transparency in reporting. For further information on Nature Research policies, see Authors & Referees and the Editorial Policy Checklist.

Statistics

For all statistical analyses, confirm that the following items are present in the figure legend, table legend, main text, or Methods section.

n/a	Confirmed	
☐	☒	The exact sample size (*n*) for each experimental group/condition, given as a discrete number and unit of measurement
☐	☒	A statement on whether measurements were taken from distinct samples or whether the same sample was measured repeatedly
☐	☒	The statistical test(s) used AND whether they are one- or two-sided<br>*Only common tests should be described solely by name; describe more complex techniques in the Methods section.*
☒	☐	A description of all covariates tested
☒	☐	A description of any assumptions or corrections, such as tests of normality and adjustment for multiple comparisons
☐	☒	A full description of the statistical parameters including central tendency (e.g. means) or other basic estimates (e.g. regression coefficient) AND variation (e.g. standard deviation) or associated estimates of uncertainty (e.g. confidence intervals)
☒	☐	For null hypothesis testing, the test statistic (e.g. *F*, *t*, *r*) with confidence intervals, effect sizes, degrees of freedom and *P* value noted<br>*Give P values as exact values whenever suitable.*
☒	☐	For Bayesian analysis, information on the choice of priors and Markov chain Monte Carlo settings
☒	☐	For hierarchical and complex designs, identification of the appropriate level for tests and full reporting of outcomes
☒	☐	Estimates of effect sizes (e.g. Cohen's *d*, Pearson's *r*), indicating how they were calculated

Our web collection on statistics for biologists contains articles on many of the points above.

Software and code

Policy information about availability of computer code

Data collection	Cell Ranger 2.2 https://support.10xgenomics.com/single-cell-gene-expression/software/pipelines/latest/installation<br>Illumina conversion software bcl2fastq2 (v2.2)-<br>Loupe Cell Browser 3.01: https://support.10xgenomics.com/single-cell-gene-expression/software/downloads/latest<br>Loupe VDJ Browser 2.01 https://support.10xgenomics.com/single-cell-gene-expression/software/downloads/latest<br>Seurat: https://satijalab.org/seurat/install.html<br>DNASEQ TCRmini - sequence analysis toolkit- available upon request. No restrictions.
Data analysis	OriginPro: https://www.originlab.com/index.aspx?go=Products/Origin<br>TCR similarity and diversity methods have been published (ref 25)<br>TCR analysis tool (available upon request, no restrictions)<br>Flow Jo v9 and 10

For manuscripts utilizing custom algorithms or software that are central to the research but not yet described in published literature, software must be made available to editors/reviewers. We strongly encourage code deposition in a community repository (e.g. GitHub). See the Nature Research guidelines for submitting code & software for further information.

Data

Policy information about availability of data

All manuscripts must include a data availability statement. This statement should provide the following information, where applicable:

- Accession codes, unique identifiers, or web links for publicly available datasets
- A list of figures that have associated raw data
- A description of any restrictions on data availability

The data discussed in this publication have been deposited in NCBI's Gene Expression Omnibus (Edgar et al., 2002) and are accessible through GEO Series accession number GSE134791 (https://www.ncbi.nlm.nih.gov/geo/query/acc.cgi?acc= GSE134791).
All other data that support the findings of this study are available from the corresponding author upon reasonable request. No restrictions on data availability

Field-specific reporting

Please select the one below that is the best fit for your research. If you are not sure, read the appropriate sections before making your selection.

☒ Life sciences ☐ Behavioural & social sciences ☐ Ecological, evolutionary & environmental sciences

For a reference copy of the document with all sections, see nature.com/documents/nr-reporting-summary-flat.pdf

Life sciences study design

All studies must disclose on these points even when the disclosure is negative.

Sample size	No statistical tests were chosen. Sample size are standard practice in the field. Sample size varies as indicated in specific experiments.
Data exclusions	No selected data was excluded from this study.
Replication	All attempts at replication were successful
Randomization	Mice were sex and age matched for examined and control samples.
Blinding	Slides with tissue sections were analyzed by an experienced clinical pathologist in a blinded manner.<br>In other experiments investigators were not blinded.

Reporting for specific materials, systems and methods

We require information from authors about some types of materials, experimental systems and methods used in many studies. Here, indicate whether each material, system or method listed is relevant to your study. If you are not sure if a list item applies to your research, read the appropriate section before selecting a response.

Materials & experimental systems

n/a	Involved in the study	
☐	☒	Antibodies
☐	☒	Eukaryotic cell lines
☒	☐	Palaeontology
☐	☒	Animals and other organisms
☒	☐	Human research participants
☒	☐	Clinical data

Methods

n/a	Involved in the study	
☒	☐	ChIP-seq
☐	☒	Flow cytometry
☒	☐	MRI-based neuroimaging

Antibodies

Antibodies used	Provided, see supplemental information
Validation	Validation statements for antibodies are available on the manufacturer's website

Eukaryotic cell lines

Policy information about cell lines

Cell line source(s)	ATCC,

Authentication	Hybridomas were authenticated by their fusion with BW5147 thymoma,resistance to HAT and flow cytometry.
Mycoplasma contamination	All lines were mycoplasma negative
Commonly misidentified lines (See ICLAC register)	N/A

Animals and other organisms

Policy information about studies involving animals; ARRIVE guidelines recommended for reporting animal research

Laboratory animals	Provided
Wild animals	N/A
Field-collected samples	The study did not involved filed-collected samples.
Ethics oversight	All experimental procedures involving mice were carried out according to protocols approved by the GSU IACUC

Note that full information on the approval of the study protocol must also be provided in the manuscript.

Flow Cytometry

Plots

Confirm that:

☒ The axis labels state the marker and fluorochrome used (e.g. CD4-FITC).

☒ The axis scales are clearly visible. Include numbers along axes only for bottom left plot of group (a 'group' is an analysis of identical markers).

☒ All plots are contour plots with outliers or pseudocolor plots.

☒ A numerical value for number of cells or percentage (with statistics) is provided.

Methodology

Sample preparation	CD4 T cells and dendritic cells were isolated as described in Methods section
Instrument	FACSCanto (BD), CytoFLEX Flow Cytometer (Beckman), MoFlo cell sorter or SH800 sorter (Sony)
Software	FlowJo (version 9 and 10) was used to analyze flowcytometry data.
Cell population abundance	Purity of sorted cells is provided in Methods section.
Gating strategy	All gating strategies have been provided in Fgure S16

☒ Tick this box to confirm that a figure exemplifying the gating strategy is provided in the Supplementary Information.
